# Trabectedin Enhances the Antitumor Effects of IL-12 in Triple-Negative Breast Cancer

**DOI:** 10.1158/2326-6066.CIR-24-0775

**Published:** 2025-01-07

**Authors:** Emily Schwarz, Himanshu Savardekar, Sara Zelinskas, Abigail Mouse, Gabriella Lapurga, Justin Lyberger, Adithe Rivaldi, Emily M. Ringwalt, Katherine E. Miller, Lianbo Yu, Gregory K. Behbehani, Timothy P. Cripe, William E. Carson

**Affiliations:** 1 The James Comprehensive Cancer Center, The Ohio State University, Columbus, Ohio.; 2 Biomedical Sciences Graduate Program, The Ohio State University, Columbus, Ohio.; 3 Division of Hematology, The Ohio State University, Columbus, Ohio.; 4 Steve and Cindy Rasmussen Institute for Genomic Medicine, Abigail Wexner Research Institute at Nationwide Children’s Hospital, Columbus, Ohio.; 5 Molecular, Cellular, and Developmental Biology Graduate Program, The Ohio State University, Columbus, Ohio.; 6 Division of Hematology, Oncology & Blood and Marrow Transplant, Center for Childhood Cancer Research, Nationwide Children’s Hospital, Columbus, Ohio.; 7 Department of Pediatrics, The Ohio State University College of Medicine, Columbus, Ohio.; 8 Center for Biostatistics, The Ohio State University, Columbus, Ohio.; 9 Pelotonia Institute for Immuno-Oncology, The James Comprehensive Cancer Center, The Ohio State University, Columbus, Ohio.; 10 Division of Surgical Oncology, The Ohio State University, Columbus, Ohio.

## Abstract

IL-12 is a potent NK cell–stimulating cytokine, but the presence of immunosuppressive myeloid cells such as myeloid-derived suppressor cells (MDSC) can inhibit IL-12–induced NK-cell cytotoxicity. Thus, we hypothesized that trabectedin, a myeloid cell–depleting agent, would improve the efficacy of IL-12 in triple-negative breast cancer (TNBC). *In vitro* treatment of healthy donor NK cells with trabectedin increased expression of the activation marker CD69 and mRNA expression of T-box transcription factor (*Tbx21*), the cytotoxic ligands TNF-related apoptosis–inducing ligand (*TNFSF10*), Fas ligand (*FASLG*), and the dendritic cell (DC)–recruiting chemokine lymphotactin (*XCL1*). The combination of IL-12 and trabectedin increased NK-cell cytotoxicity and activation and production of IFN-γ, TNF-α, and granzyme B in the presence of human TNBC cells. Treatment of 4T1 and EMT6 tumor–bearing mice with IL-12 and trabectedin led to a significant reduction in tumor burden compared with single-agent controls and the highest levels of plasma IFN-γ, intratumoral CD8^+^ T cells, and conventional type 1 DC. MDSC and M2-like macrophages were significantly decreased with combination therapy. NK-cell depletion abrogated the effects of combination therapy, as did the elimination of CD8^+^ T cells. NK-cell depletion led to lower levels of the NK cell–derived chemokine CCL5 and the DC-derived chemokine CXCL10, higher tumor burden, and decreased intratumoral CD8^+^ T cells. IL-12 and trabectedin also significantly enhanced the response of TNBC to anti–PD-L1 therapy. These data suggest that MDSC depletion augments the ability of IL-12–activated NK cells to drive the infiltration of DC and CD8^+^ T cells into TNBC for an antitumor effect.

## Teaser

TNBC is associated with a poor prognosis. The authors show that adding trabectedin to IL-12 is effective in murine TNBC models as it augments the effects of IL-12–activated NK cells, suggesting new potential treatment options for TNBC.

## Introduction

IL-12 is a proinflammatory cytokine capable of inducing innate and adaptive immune responses (1). The ability of IL-12 to activate cytotoxic NK cells and T cells led to its identification as a potential immunotherapeutic agent. IL-12 therapy has demonstrated moderate efficacy in several tumor types, including HER2-positive breast cancer, melanoma, and renal cell carcinoma, but questions remain about the optimal dose in relation to the toxicity profile (2, 3). In the end, most patients fail to respond to IL-12 therapy (4). Several methods have been used to improve the efficacy of IL-12, including localized administration techniques, gene therapy strategies, and transfer of IL-12–transduced autologous cells (3).

In an effort to elucidate potential mechanisms of resistance to IL-12, several groups have conducted analyses of patients who received IL-12 therapy (4–6). In a clinical trial of IL-12 with trastuzumab, an HER2-targeted mAb, sustained IFN-γ production by NK cells, was detected only in patients who experienced clinical benefit (5). Similar results were seen in a follow-up trial combining IL-12 with trastuzumab and the chemotherapeutic paclitaxel, in which only patients who had clinical benefit had measurable IFN-γ levels (2). In that study, IFN-γ output was also significantly associated with increased progression-free survival and observed solely in NK cells. Preclinical models showed that the depletion of NK cells prior to IL-12 administration attenuated its antitumor effect (7). These results suggest that the NK-cell response to IL-12 therapy may be an essential element for achieving clinical benefit.

In some solid tumors, such as breast cancer, the presence of a highly immunosuppressive tumor microenvironment (TME) may prevent the NK-cell response to IL-12 (8). About 15% of breast cancers lack expression of estrogen, progesterone, and HER2/neu receptors [so-called triple-negative breast cancer (TNBC); ref. 9]. TNBC is one of the most difficult-to-treat types of breast cancer because of its lack of targetable receptor expression coupled with its immunosuppressive TME. Currently, only cytotoxic chemotherapy is available to patients with advanced disease (10). The incidence and outcome of TNBC are also highly disproportionate, with Black women twice as likely to be affected and more likely to die from the disease than White women (11). Thus, there is an increasing need to develop additional therapeutic options for TNBC.

One of the populations in the TME that can inhibit NK cells is myeloid-derived suppressor cells (MDSC), which are increased in TNBC compared with non-TNBC and correlate with a worse prognosis (12, 13). TNBC can also exhibit tumor-induced dysfunctional NK cells (14). MDSC have been shown to significantly inhibit NK-cell cytotoxicity and cytokine production, two critical antitumor effector mechanisms augmented by IL-12 (15). MDSC can impair NK cells via contact-dependent mechanisms such as inhibitory ligand expression and through contact-independent mechanisms such as production of TGF-β, arginase, and reactive oxygen species (16, 17). Our group has shown that MDSC-derived nitric oxide can antagonize Fc receptor–mediated NK-cell functions and response to antibody therapy (15). Moreover, reduced percentages of circulating MDSC have been found to correlate with improved progression-free survival in IL-12–treated patients (18). Furthermore, monocytic MDSC (M-MDSC) can differentiate into M2-like tumor-associated macrophages (TAM) within the TME, and increased levels of TAMs are also associated with poor prognosis in TNBC (19). Thus, MDSC may mediate one mechanism preventing responses to IL-12 therapy.

Trabectedin is an FDA-approved therapeutic that can inhibit MDSC and M2-like TAMs (20). It has been utilized for the treatment of sarcoma because of its DNA-alkylating abilities; however, it has also been shown to be capable of mimicking the TNF-related apoptosis–inducing ligand (TRAIL; ref. 20). TRAIL activation stimulates caspase-8–dependent apoptosis in TRAIL receptor 1/2–positive cells. MDSC and TAMs have increased TRAIL receptor 1/2 expression compared with lymphocytes and polymorphonuclear cells (PMN), resulting in a selective reduction in these populations by trabectedin (21). The myelolytic effect of trabectedin has been demonstrated in several models of cancer (22, 23).

The present study sought to determine whether trabectedin would enhance the antitumor activity of IL-12. To test this, the impact of trabectedin on myeloid and NK cells was examined *in vitro* and in murine models of TNBC.

## Materials and Methods

### Cell lines

Murine TNBC cell lines 4T1 (ATCC Cat. #CRL-2539, RRID: CVCL_0125) and EMT6 (ATCC Cat. #CRL-2755, RRID: CVCL_1923), human chronic myelogenous leukemia cell line K562 (ATCC Cat. #CRL-3343, RRID: CVCL_UC15), and human TNBC cell line MDA-MB-468 (ATCC Cat. #HTB-132, RRID: CVCL_0419) were purchased from ATCC in 2010 (4T1 and EMT6), 2014 (K562), and 2015 (MDA-MB-468). Human TNBC cell lines MDA-MB-231 (RRID: CVCL_0062) and MDA-MB-436 (RRID: CVCL_0623) were gifts from the laboratory of Dr. Steven Sizemore (The Ohio State University) in 2023. All cell lines were maintained in culture with media [RPMI 1640: 4T1, EMT6, K562, and MDA-MB-231 (Thermo Fisher Scientific Cat. #22400105) or DMEM: MDA-MB-468 and MDA-MB-436 (Life Technologies Cat. #11965-118)] supplemented with 10% heat-inactivated FBS (Sigma-Aldrich Cat. #F4135) and 1% antibiotic–antimycotic (Thermo Fisher Scientific Cat. #15240-062). Each individual cell line was confirmed to be *Mycoplasma* free by internal testing twice monthly using the Mycoplasma Detection Kit (SouthernBiotech Cat. #OB1310001), authenticated by cytogenetic/karyotyping analysis and used for no more than 20 passages.

### Human NK-cell isolation and treatment

Human peripheral blood mononuclear cells (PBMC) were obtained from healthy donor buffy coats acquired as commercially available products from Versiti Blood Center of Ohio. Each donor provided written informed consent prior to sample acquisition. Buffy coat samples from 30 healthy donors were included in the experimental studies under institutional review board–approved protocol for human subject research (OSU IRB protocol 1999C0348). Samples were shipped overnight at room temperature and processed immediately upon receipt. PBMCs were isolated from buffy coats using a Ficoll-Hypaque density gradient centrifugation technique, as described (15). Briefly, buffy coat samples were layered on the top of the Ficoll density gradient medium (density = 1.077 g/mL) and centrifuged at 1,500 rpm for 25 minutes with the brake off. PBMCs were removed from the density gradient medium, and residual red blood cells (RBC) were lysed using RBC lysis buffer (Cell Signaling Technology Cat. #46232). NK cells were isolated from PBMCs using the EasySep Human NK cell Isolation Kit (STEMCELL Technologies Cat. #17955) according to the manufacturer’s protocol. Human NK cells were maintained in culture with RPMI 1640 media (Thermo Fisher Scientific Cat. #22400105) supplemented with 10% heat-inactivated human AB serum (Sigma-Aldrich Cat. #H4522) and 1% antibiotic–antimycotic (Thermo Fisher Scientific Cat. #15240-062). Human NK cells used in downstream assays were treated *in vitro* with (<0.01%) DMSO (Sigma-Aldrich Cat. #D2650-100ML), 10 ng/mL recombinant human IL-12 (R&D Systems Cat. #219-IL-005), and/or 2.5 nmol/L trabectedin (MedChemExpress Cat. #HY-50936).

### Flow cytometry

Purity of isolated human NK cells was confirmed using fluorescently labeled PerCP-Cy5.5 anti-human CD56 (BioLegend Cat. #398811, RRID: AB_2894512) and Pacific Blue anti-human CD3 (BioLegend Cat. #300329, RRID: AB_10552893) antibodies. Samples were used in downstream analyses if purity of NK cells (CD3^−^CD56^+^) was at least 90%. To assess treatment-induced cellular apoptosis, NK cells were treated *in vitro* for 48 hours, harvested, and stained with allophycocyanin APC anti-human/mouse/rat annexin V (BD Biosciences Cat. #561012, RRID: AB_2034024) and PE propidium iodide staining solution (BD Biosciences Cat. #556463, RRID: AB_2869075). Cells negative for annexin V and propidium iodide were considered viable. Activation of NK cells *in vitro* was assessed by surface expression of CD69 using APC anti-human CD69 (BioLegend Cat. #310910, RRID: AB_314845) at 24 hours. STAT4 phosphorylation was measured by intracellular flow cytometry using PE anti-human STAT4 phospho antibody (BioLegend Cat. #941205, RRID: AB_2936724) after 2 hours treatment *in vitro* followed by fixation and permeabilization using True-Phos Perm Buffer (BioLegend Cat. #425401). For the measurement of murine splenic MDSC, spleens were processed into single-cell suspensions, lysed for RBCs using RBC lysis buffer (Cell Signaling Technology Cat. #46232), and stained with PE anti-mouse CD11b (BioLegend Cat. #101208, RRID: AB_312791), Pacific Blue anti-mouse Ly6C (BioLegend Cat. #128032, RRID: AB_2562178), and APC anti-mouse Ly6G (BioLegend Cat. # 127614, RRID: AB_2227348). Total MDSC were defined as CD45^+^CD11b^+^Ly6C^±^Ly6G^±^, M-MDSC were defined as CD45^+^CD11b^+^Ly6C^+^, and PMN-MDSC were defined as CD45^+^CD11b^+^Ly6G^+^ (17). All flow cytometric samples were analyzed using an LSRFortessa flow cytometer (BD Biosciences), and a minimum of 10,000 events were recorded per sample. FlowJo v10.6.1 (FlowJo, RRID: SCR_008520) was used for gating and analysis.

### RT-PCR

RNA was isolated from *in vitro* treated human NK cells using the mirVana isolation kit (Thermo Fisher Scientific Cat. #AM1560). A NanoDrop spectrophotometer (Thermo Fisher Scientific) was used to assess the purities and concentrations of RNA. Reverse transcription was performed using 500 ng RNA in a 20 μL reaction with the high-capacity cDNA Reverse Transcription Kit (Thermo Fisher Scientific Cat. #4368814) to isolate cDNA. cDNA was then used to measure gene expression using SYBER Green (Integrated DNA Technologies) or TaqMan (Thermo Fisher Scientific) chemistry for qRT-PCR with the following commercially available human primers: *ACTB* (used for normalization, Hs.PT.39a.22214847, Integrated DNA Technologies), *T-BET* (T-box transcription factor; Hs.PT.58.3936407, Integrated DNA Technologies), *TNFSF10* (Hs00234355_m1, Thermo Fisher Scientific), *FASLG* (Hs01904942_s1, Thermo Fisher Scientific), *XCL1* (Hs.PT.58.26896865.g, Integrated DNA Technologies), *IFNG* (Hs.PT.58.3781960, Integrated DNA Technologies), and *GZMB* (Hs00188051_m1, Thermo Fisher Scientific). Each RT-PCR reaction was performed in triplicate using the ABI PRISM 7900HT Fast RT-PCR system (Applied Biosystems), and the 2^ΔΔCt^ method was used to calculate fold changes. Statistical analyses were performed in GraphPad Prism v10 (RRID: SCR_002798) using a one-way repeated measures ANOVA (RM one-way ANOVA) with the Tukey multiple comparisons test.

### Cytotoxicity assay

Isolated human NK cells were pretreated *in vitro* prior to use with (<0.01%) DMSO (Sigma-Aldrich Cat. #D2650-100ML), 10 ng/mL recombinant human IL-12 (R&D Systems Cat. #219-IL-005), and/or 2.5 nmol/L trabectedin (MedChemExpress Cat. #HY-50936). After 24 hours, NK cells were harvested and resuspended in fresh RPMI media supplemented with 20% heat-inactivated FBS and 1% antibiotic–antimycotic. Untreated tumor cells were labeled with carboxyfluorescein succinimidyl ester (Cat. #C34554, Thermo Fisher Scientific) and resuspended in the same media. NK cells from each treatment condition and tumor cells were then co-cultured at an effector to target ratio of 10:1. After a 4-hour incubation, co-culture samples were harvested and stained using APC anti-human/mouse/rat annexin V antibody (BD Biosciences Cat. #561012, RRID: AB_2034024) and SYTOX Blue dead cell stain (Thermo Fisher Scientific Cat. #S34857). A minimum of 10,000 tumor cell events were collected per sample using an LSRFortessa flow cytometer (BD Biosciences). Specific tumor cell lysis was calculated after subtracting the percentage of spontaneously lysed tumor cells in the negative controls (wells with only tumor cells) from the percentage of lysed tumor cells in each condition. Statistical analyses were performed in GraphPad Prism v10 (RRID: SCR_002798) using RM one-way ANOVA with the Tukey multiple comparisons test or paired student *t* tests when appropriate.

### Degranulation assay

NK-cell degranulation was measured via flow cytometric evaluation of CD107a expression on the NK-cell surface after incubation with K562 cells. Briefly, NK cells were pretreated for 24 hours, harvested, resuspended in fresh media, and combined in a co-culture with K562 cells at an effector to target ratio of 10:1. Carboxyfluorescein succinimidyl ester (Thermo Fisher Scientific Cat. #C34554) labeling was used to distinguish tumor cells from NK cells. Three microliters of PE anti-human CD107a (BioLegend Cat. #328607, RRID: AB_1186062) was immediately added to each well after cells were combined. After a 1-hour incubation, 1× Protein Transport Inhibitor Cocktail (eBioscience Cat. #00-4980-93) was added to each well, and incubation was continued for another 3 hours. Samples were harvested and analyzed by flow cytometry for CD107a expression using an LSRFortessa flow cytometer (BD Biosciences). A minimum of 10,000 NK cell events were recorded for all samples. Statistical analyses were performed in GraphPad Prism v10 (RRID: SCR_002798) using RM one-way ANOVA with the Tukey multiple comparisons test.

### ELISA

NK cells were pretreated for 24 hours, harvested, resuspended in fresh media, and combined in culture with MDA-MB-436 cells at an effector to target ratio of 10:1. After 24 hours, cell-free supernatants were assessed for levels of IFN-γ (R&D Systems Cat. #DY285B-05), TNF-α (R&D Systems Cat. #DY210-05), and granzyme B (R&D Systems Cat. #DGZB00) by ELISAs according to the manufacturer’s instructions. Statistical analyses were performed in GraphPad Prism v10 (RRID: SCR_002798) using paired student *t* tests. For murine IFN-γ levels, plasma was analyzed using a mouse IFN-γ Quantikine ELISA Kit (R&D Systems Cat. #MIF00) according to the manufacturer’s instructions. Statistical analyses were performed in GraphPad Prism v10 using RM one-way ANOVA with the Tukey multiple comparisons test.

### Murine tumor models

To establish orthotopic murine models of TNBC, 4- to 6-week-old female BALB/c mice (The Jackson Laboratory, RRID: MGI:2683685) were inoculated with 1 × 10^5^ 4T1 cells or 1 × 10^6^ EMT6 cells in the mammary fat pad. Once tumors reached at least 50 mm^3^, mice were divided equally and randomly into treatment groups. Recombinant mouse IL-12 (rmIL-12; R&D Systems Cat. #419-ML-050/CF) was reconstituted in sterile PBS and stored at −80°C. On the day of injection, rmIL-12 was thawed and diluted in sterile PBS to 0.5 μg per 100 μL. rmIL-12 was administered by intraperitoneal injection 3×/week in a final volume of 100 μL. Trabectedin (MedChemExpress Cat. #HY-50936) was dissolved in DMSO at 4 mg/mL and stored at −20°C. On the day of injection, trabectedin was thawed and diluted in 0.1 g of hydroxy B-cyclodextrin (Sigma-Aldrich Cat. #332607-5G) and sterile PBS. Trabectedin was administered by tail vein injection 1×/week in a final volume of 100 μL at 0.15 mg/kg. Control treatments were performed with matched intraperitoneal or tail vein injections of 100 µL PBS. NK-cell depletion was performed through the administration of 100 μL of 1 mg/mL polyclonal anti–asialo-GM1 (FUJIFILM Wako Shibayagi Cat. #986-10001, RRID: AB_516844) via intraperitoneal injection 3 days prior to the start of treatment and every 4 days thereafter. Anti–asialo-GM1 was reconstituted in distilled water upon receipt, and dilutions were performed using PBS. This antibody has been used to perform *in vivo* depletion of NK cells in mouse strains lacking the NK1.1 allotype, which is a feature of BALB/c mice (14). We have previously determined that this regimen removes the majority of NK cells while leaving the macrophage compartment largely unaffected (24). A measure of 100 μL of IgG isotype control antibody (Bio X Cell Cat. #BE0095, RRID: AB_1107793) was injected intraperitoneally into nondepleted mice at the same time points. CD8a depletion was performed through intraperitoneal injection of 100 μL anti-CD8a (Bio X Cell Cat. #BE0061, RRID: AB_1125541) 1 day prior to the start of treatment (at a dose of 200 μg) and every 4 days thereafter (at a dose of 100 μg). Anti-CD8a dilutions were performed using PBS. A measure of 100 μL of IgG isotype control antibody (Bio X Cell Cat. #BE0095, RRID: AB_1107793) was injected intraperitoneally into control mice at the same time points. Anti–PD-L1 (Bio X Cell Cat. #BE0101, RRID: AB_10949073) was administered by intraperitoneal injection (100 μg/100 μL) 3×/week. An IgG isotype control antibody (100 μL, Bio X Cell Cat. #BE0095, RRID: AB_1107793) was used as the control for anti–PD-L1 therapy and given intraperitoneally at a dose of 100 μg/100 μL 3×/week. Tumor volume measurements were performed 3×/week using digital calipers for all studies. Mouse weights were assessed throughout treatments using a digital scale. All murine studies were approved by The Ohio State University Institutional Animal Care and Use Committee and conducted under an approved protocol (IACUC 2009A0179-R2). For statistical analyses of tumor volumes, a linear mixed modeling was used to model longitudinal tumor volume for mice under each treatment. Comparisons were done at each time point and averaged across all time points using t-statistics. The Tukey–Kramer method was used for adjusting raw *P* values for multiple comparisons across treatment groups.

### Mass cytometry

To evaluate splenic and/or tumor immune cell infiltrates in mice after treatment, a 30-antibody cytometry by time-of-flight panel was used (detailed reagent specifications in Supplementary Table S1), as previously described (25). In brief, spleens and tumors were processed into single-cell suspensions, washed with Maxpar Cell Staining Buffer (CSB; Standard BioTools Cat. #201068), and incubated for 10 minutes in Fc-blocking solution (BioLegend Cat. #422301). Cells were stained with 45 μL of surface antibody cocktail and incubated at 4°C for 30 minutes before fixation with 1.4× Proteomic Stabilizer PROT1 buffer (Smart Tube Inc. Cat. #501351689) at −80°C. For intracellular staining, cells were washed with CSB and incubated for 15 minutes in methanol at −20°C. Samples were incubated with 50 μL of intracellular antibody cocktail for 50 minutes before 1.5% paraformaldehyde (Thermo Fisher Scientific Cat. #28906) and 500 μmol/L Ir intercalator (Standard BioTools Cat. # 201103A) were added. A 30-minute incubation at 37°C was performed, followed by washes with CSB and H_2_O prior to data acquisition. Samples were analyzed using cloud-based computing software Cytobank v10.2 (RRID: SCR_014043). Immune population and phenotype definitions are described in Supplementary Table S2. Statistical analyses were performed in GraphPad Prism v10 (RRID: SCR_002798) using ordinary one-way ANOVA tests with Tukey correction to adjust for multiple comparisons.

### NanoString digital spatial profiling of murine tumors

Formalin-fixed, paraffin-embedded mouse tumor tissue sections (5 μm thickness) were prepared on Superfrost Plus slides (*n* = 2 tumor tissue sections per slide, 12 slides total) according to the GeoMx Digital Spatial Profiler (DSP) protocol (NanoString Technologies, Inc., NanoString GeoMx DSP Manual Slide Preparation; MAN-10150-01). Three slides per treatment condition were analyzed (12 slides total and 24 total tumor tissue sections). Tissue preparation, antigen retrieval, and antibody staining were performed, as previously described (26). Fluorescently tagged antibodies used for staining were the nuclear stain SYTO13 (121300303, NanoString Technologies, Inc.), leukocyte marker CD45-EM-05 (NanoString Technologies, Inc., NBP1-44763), tumor cell marker PanCK-AE1 + PanCK-AE3 (NanoString Technologies, Inc., NBP2-33200), and NK-cell marker CD49b-PerCP/Cyanine5.5 (BioLegend, clone HMα2, 1:50 dilution). The following oligo-tagged NanoString GeoMx mouse detection antibody mixtures were used: Immune Cell Profiling Panel, Cell Death Panel, IO Drug Target Panel, Immune Activation Status Panel, Immune Cell Typing Panel, PI3K/AKT Signaling Panel, and MAPK Signaling Panel Death quantifying expression of a total of 71 proteins (see Supplementary Table S3 for the detailed list of detection targets and antibodies).

Stained slides were scanned using a GeoMx DSP instrument (NanoString Technologies, Inc.). Six regions of interest (ROI) were selected within each tumor tissue section for a total of 12 ROIs per slide (*n* = 3 slides/treatment condition). A total of 144 ROIs were analyzed. In each ROI, immune cells and tumor cells were defined by manually selecting a CD45 fluorescence threshold and PanCK fluorescence threshold that visually maximized the two individual cell types. Oligos from each ROI segment were cleaved, collected, and dried in a 96-well flat-bottom plate. Oligos were subsequently hybridized to unique reporter tags and counted using the nCounter platform (NanoString Technologies, Inc.) according to the manufacturer’s protocol. GeoMx software v2.4 (NanoString Technologies, Inc.) was used for all data quality control, normalization, and differential expression analyses according to the manufacturer’s recommendations. Proteins significantly differentially expressed between treatment groups were determined using a linear mixed model to account for multiple ROI sections within individual samples and batch effects between runs followed by *P* value correction using the Benjamini–Hochberg procedure.

### Mouse Luminex Discovery Assay

Plasma was isolated from mice at the time of sacrifice for both the NK-cell depletion and CD8a depletion studies 24 hours after IL-12 administration. A bead-based Luminex Multiplex Assay (R&D Systems LXSAMSM-10 lot #L151841) was then used to measure circulating levels of IFN-γ (BR33), TNF-α (BR14), GM-CSF (BR12), granzyme B (BR63), CCL2 (BR18), CCL3 (BR46), CCL4 (BR51), CCL5 (BR38), CCL22 (BR75), and CXCL10 (BR37). Data acquisition and analysis were performed using a Luminex MAGPIX platform with the xPONENT 4.3 program (R&D Systems). Data were reported only if measurable cytokine levels were detected in at least three mice/condition. Statistical analyses were performed in GraphPad Prism v10 (RRID: SCR_002798) using an unpaired two-tailed student *t* test.

### IHC

IHC analysis of mouse tumors was performed by HistoWiz Inc. (histowiz.com) using standard procedures and an automated workflow (27). Briefly, tumors were excised and immediately fixed with neutral-buffered 10% formalin for 24 to 48 hours and then stored in PBS at 37°C. Samples were processed, embedded in paraffin, and sectioned onto Superfrost Plus slides at 5 μmol/L thickness. IHC was then performed on a BOND RX autostainer (Leica Biosystems) with enzyme treatment (1:1,000 dilution) using HistoWiz standard protocols. Antibodies used were rabbit monoclonal CD4 primary antibody (Abcam Cat. #ab183685, RRID: AB_2686917, 1:500 dilution), rabbit monoclonal CD8a primary antibody (Cell Signaling Technology Cat. #98941, RRID: AB_2756376, 1:600 dilution), and rabbit anti-rat secondary antibody (Vector Laboratories Cat. #AI-4001, RRID: AB_2336209, 1:100 dilution). BOND Polymer Refine Detection was utilized following the manufacturer’s protocol (Leica Biosystems). After staining was completed, tumor sections were dehydrated and coverslipped with film using a Tissue-Tek Prisma and Coverslipper (Sakura). Slide scanning was performed at 40× magnification on Aperio AT2 (Leica Biosystems). Quantitative analysis of CD4- and CD8a-positive staining was performed using the open-source digital image analyzer QuPath v0.4.3 (RRID: SCR_018257). Data were analyzed in GraphPad Prism v10 (RRID: SCR_002798) using two-tailed unpaired student *t* tests for statistical analysis.

### Statistics

Statistical analyses of three or more treatment groups were performed using one-way ANOVA tests with Tukey correction to adjust for multiple comparisons unless otherwise noted. Statistical analyses between two groups were performed with unpaired two-tailed student *t* tests unless noted otherwise. ANOVA tests with Tukey correction and student *t* tests were conducted using GraphPad Prism v10 (RRID: SCR_002798). For statistical analyses of murine tumor volumes, linear mixed modeling was used to model longitudinal tumor volume for mice under each treatment. Comparisons were done at each time point and averaged across all time points using t-statistics. The Tukey–Kramer method was used for adjusting raw *P* values for multiple comparisons across treatment groups.

### Data availability

All data generated during this study are available in the published article, and its Supplementary data files can be made available upon reasonable request to the corresponding author.

## Results

### IL-12 and trabectedin treatment increases NK-cell activation *in vitro*

**Figure 1.**
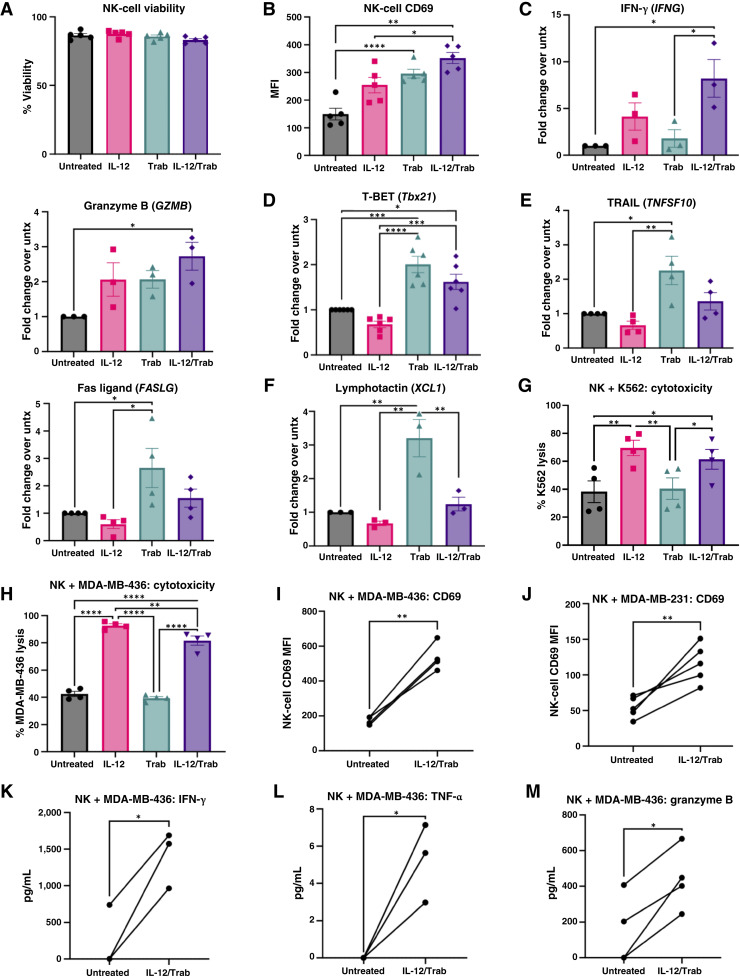
IL-12 and trabectedin treatment increases human NK-cell activation *in vitro*. **A,** NK-cell viability after 48-hour treatment with DMSO (control), 10 ng/mL IL-12, 2.5 nmol/L trabectedin, or IL-12 plus trabectedin (*n* = 5 donors). **B,** Surface CD69 expression on NK cells after 24-hour treatment. Values are mean fluorescence intensity (MFI; *n* = 5 donors). The mRNA expression of (**C**) *IFNG*, *GZMB*, (**D**) *Tbx21*, (**E**) *TNFSF10*, *FASLG*, and (**F**) *XCL1* in NK cells after 24-hour treatment. Values show fold changes in mRNA expression compared with respective DMSO-treated NK cells (untx; *n* = 3–6 donors). **G,** K562 tumor cell lysis after a 4-hour NK-cell cytotoxicity assay (*n* = 4 donors). **H,** MDA-MB-436 tumor cell lysis after a 4-hour NK-cell cytotoxicity assay (*n* = 4 donors). Statistical analyses were performed using RM one-way ANOVA with the Tukey multiple comparisons test. Data represent mean ± SEM. Surface CD69 expression on NK cells following a 4-hour co-culture with (**I**) MDA-MB-436 cells (*n* = 4 donors) or (**J**) MDA-MB-231 cells (*n* = 5 donors). Quantification of (**K**) IFN-γ (*n* = 3 donors), (**L**) TNF-α (*n* = 3 donors), and (**M**) granzyme B (*n* = 4 donors) secretion by pretreated NK cells after 24-hour co-culture with MDA-MB-436 cells. Statistical analyses were performed using paired student *t* tests. *, *P* < 0.05; **, *P* < 0.01; ***, *P* < 0.001; ****, *P* < 0.0001. Trab, trabectedin.

Although the NK-cell response to IL-12 has been extensively characterized, the direct effects of trabectedin on NK cells remain unknown. Human NK cells were isolated (purity gating in Supplementary Fig. S1A) and treated for 24 hours *in vitro* with DMSO control, 10 ng/mL IL-12, 2.5 nmol/L trabectedin, or IL-12 and trabectedin, with dosing based on previous studies (28). NK-cell viability was unaffected by IL-12 and/or trabectedin (Fig. 1A), confirming that the myeloid cell–specific toxicity of trabectedin did not extend to NK cells. CD69 expression was measured as an initial marker of NK-cell activation. Trabectedin alone induced CD69 expression (*P* < 0.001 vs. control), and this was further increased when IL-12 and trabectedin were combined (*P* = 0.005 vs. control; Fig. 1B). Subsequently, the expression of several NK-cell activation and cytotoxicity genes was evaluated by RT-PCR. Trabectedin and IL-12 combined to induce the highest expression of IFN-γ (*IFNG*) and granzyme B (*GZMB*) in NK cells (all *P* < 0.05 vs. control; Fig. 1C), exceeding the level induced by either agent alone. Trabectedin alone significantly increased the expression of T-BET (*Tbx21*), a transcription factor important for cytotoxicity (Fig. 1D), as well as cytotoxic ligands TRAIL (*TNFSF10*) and Fas ligand (*FASLG*; Fig. 1E), and lymphotactin (*XCL1*; Fig. 1F), a chemokine important for mediating dendritic cell (DC) migration (all *P* < 0.05 vs. control and IL-12). As STAT4 is a known mediator of IL-12–induced NK-cell signaling, STAT4 phosphorylation was measured in NK cells treated with IL-12, trabectedin, or the combination (1). STAT4 phosphorylation was significantly increased in IL-12–treated NK cells compared with control-treated NK cells (*P* = 0.02; Supplementary Fig. S1B). Phospho-STAT4 levels were similar between IL-12 and combination-treated NK cells (*P* = 0.048 for combination vs. control), whereas trabectedin alone had little effect. NK cells were then tested for cytotoxicity against K562 tumor cells (gating strategy for tumor cell lysis in Supplementary Fig. S1C). NK-cell cytotoxicity was significantly increased following IL-12 treatment (*P* = 0.003 vs. control; Fig. 1G). Although trabectedin alone had minimal effects, trabectedin in combination with IL-12 significantly increased NK-cell cytotoxicity (both *P* < 0.03; Fig. 1G) and degranulation against K562 cells (both *P* < 0.05; Supplementary Fig. S1D) compared with control and single-agent trabectedin. NK-cell cytotoxicity was then measured against human TNBC cell lines MDA-MB-436, MDA-MB-231, and MDA-MB-468. Compared with control, IL-12 and IL-12 plus trabectedin significantly increased NK-cell cytotoxicity against MDA-MB-436 and MDA-MB-231 cells (both *P* < 0.01; Fig. 1H; Supplementary Fig. S1E). IL-12 and IL-12 plus trabectedin also significantly increased NK-cell cytotoxicity against MDA-MB-468 cells compared with trabectedin alone (both *P* < 0.05; Supplementary Fig. S1F). Single-agent trabectedin had little effect on NK-cell cytotoxicity against these cell lines; however, the lack of an inhibitory effect by trabectedin is notable and represents new information. Pretreated NK cells cultured with MDA-MB-436 cells also had significantly higher CD69 expression, indicating increased activation in the presence of TNBC cells (*P* = 0.002; Fig. 1I). This significant increase in activation was also seen with MDA-MB-231 cells (*P* = 0.01; Fig. 1J). To evaluate NK-cell cytokine production, pretreated NK cells were cultured with MDA-MB-436 cells for 24 hours, and the supernatants were assayed. Combination-treated NK cells produced significantly higher levels of IFN-γ (*P* = 0.03; Fig. 1K), TNF-α (*P* = 0.049; Fig. 1L), and granzyme B (*P* = 0.01; Fig. 1M) than control-treated NK cells. Taken together, these data suggest that trabectedin has a direct stimulatory effect on NK cells and can be combined with IL-12 to activate NK cells in the presence of TNBC.

### Combination IL-12 and trabectedin reduces TNBC tumor burden *in vivo*

**Figure 2.**
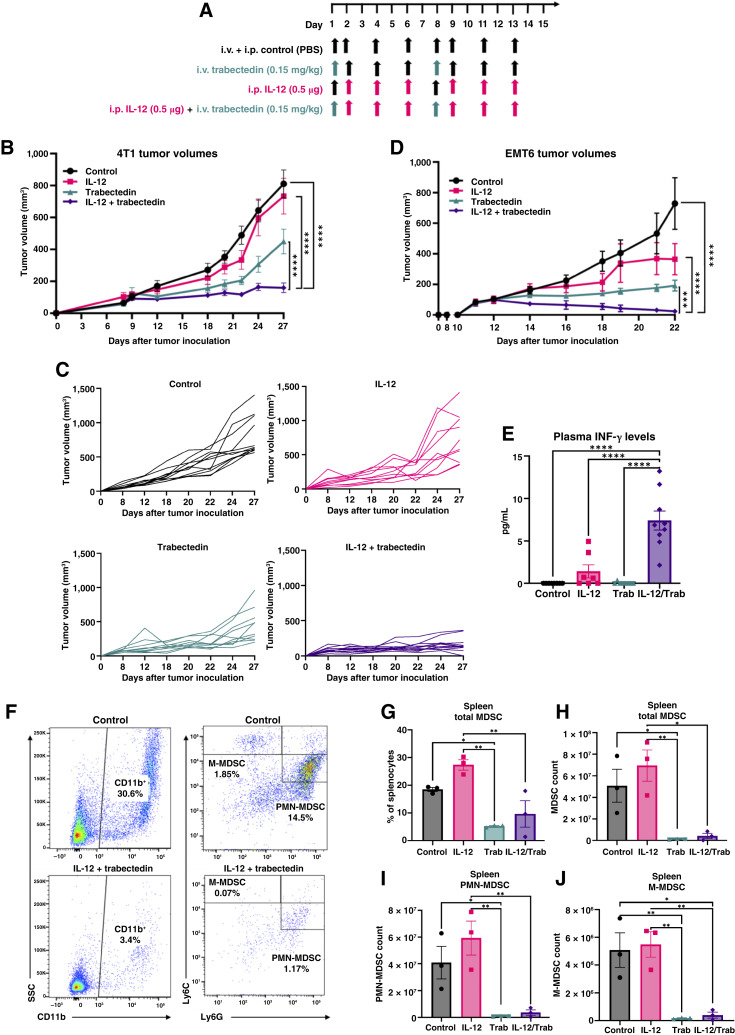
Combination IL-12 and trabectedin effectively reduces TNBC tumor burden. **A,** General treatment schema for murine studies. PBS was used as the control. IL-12 (0.5 μg) was given intraperitoneally 3×/week and 0.15 mg/kg trabectedin was given intravenously 1×/week. **B,** BALB/c mice were inoculated with 4T1 cells and divided into one of four treatment groups once tumors reached ∼50 mm^3^ [PBS control (*n* = 11), IL-12 (*n* = 10), trabectedin (*n* = 10), and IL-12 plus trabectedin (*n* = 12)]. Tumor growth was measured for 15 days. **C,** Individual tumor growth curves of mice treated with PBS control (*n* = 11), IL-12 (*n* = 10), trabectedin (*n* = 10), and IL-12 plus trabectedin (*n* = 12). **D,** Tumor growth curves of BALB/c mice inoculated with EMT6 cells and treated for 11 days, as described in (**A**) [PBS control (*n* = 7), IL-12 (*n* = 5), trabectedin (*n* = 7), and IL-12+ trabectedin (*n* = 6)]. **E,** Plasma IFN-γ levels in 4T1 tumor–bearing mice at day 15 (*n* = 7–9/treatment group). **F,** Representative flow cytometry gating for M-MDSC and PMN-MDSC from splenocytes of control-treated and IL-12 and trabectedin–treated 4T1 tumor–bearing mice. **G,** Percent MDSC in the spleens of 4T1 tumor–bearing mice 15 days after treatment (*n* = 3). **H,** Absolute counts of total MDSC and (**I**) PMN-MDSC and (**J**) M-MDSC subsets in the spleens of 4T1 tumor–bearing mice 15 days after treatment (*n* = 3). For statistical analyses of tumor volumes, linear mixed modeling was used to model the longitudinal tumor volume for mice under each treatment. Comparisons were done at each time point and averaged across all time points using t-statistics. The Tukey–Kramer method was used for adjusting raw *P* values for multiple comparisons across treatment groups. An ordinary one-way ANOVA with the Tukey multiple comparisons test was used for statistical analysis of bar graphs. Data represent mean ± SEM. *, *P* < 0.05; **, *P* < 0.01; ***, *P* < 0.001; ****, *P* < 0.0001. SSC, side scatter; Trab, trabectedin.

Given the role of trabectedin in reducing suppressive myeloid cells and the role of IL-12 in stimulating NK cells, we hypothesized that these agents would combine effectively to control tumor burden *in vivo*. Also, the above experiments indicated that these two agents might have combinatorial effects on the NK-cell compartment in a murine model of TNBC. The 4T1 model of TNBC was used to test this hypothesis as these tumors exhibits high levels of MDSC and impaired NK-cell immunity (29, 30). 4T1 tumor–bearing mice were treated for 15 days with either PBS control, IL-12 thrice weekly, trabectedin once weekly, or a combination of IL-12 and trabectedin at doses consistent with those used in prior studies by our group (Fig. 2A; ref. 23). The combination of IL-12 and trabectedin significantly reduced 4T1 tumor growth compared with single-agent IL-12 (*P* < 0.0001) and single-agent trabectedin (*P* = 0.0002; Fig. 2B and C). There was also one complete response in the IL-12 and trabectedin combination group. No significant changes in body weight were detected in any group during this time frame, suggesting limited treatment-induced toxicity (Supplementary Fig. S2A and S2B). The EMT6 model of TNBC was used to confirm the efficacy of combination therapy. In mice bearing EMT6 tumors and receiving the same doses of IL-12 and/or trabectedin as the 4T1 tumor–bearing mice, the tumor volume was significantly reduced with combination therapy compared with the single agents or control (all *P* < 0.0005; Fig. 2D). There were also three complete responses in the combination group. Circulating IFN-γ was measured in 4T1 tumor–bearing mice, given the role of IFN-γ in mediating responses to IL-12 (2). Consistent with this concept, plasma IFN-γ levels were significantly higher in combination-treated mice than in IL-12–treated mice (*P* < 0.0001), whereas control- and trabectedin-treated mice had no measurable IFN-γ in circulation 72 hours after IL-12 administration (Fig. 2E). Splenic MDSC levels were also measured to evaluate the effects on this immunosuppressive population (Fig. 2F). Both trabectedin and IL-12 plus trabectedin significantly reduced the percentage of MDSC compared with single-agent IL-12 and control treatments (all *P* < 0.04; Fig. 2G). The total counts of MDSC and PMN-MDSC and M-MDSC subsets were also significantly lower following treatment with trabectedin or IL-12 plus trabectedin (all *P* < 0.05; Fig. 2H–J).

### Combination IL-12 and trabectedin therapy reduces immunosuppressive myeloid cells while increasing intratumoral CD8^+^ T cells

**Figure 3.**
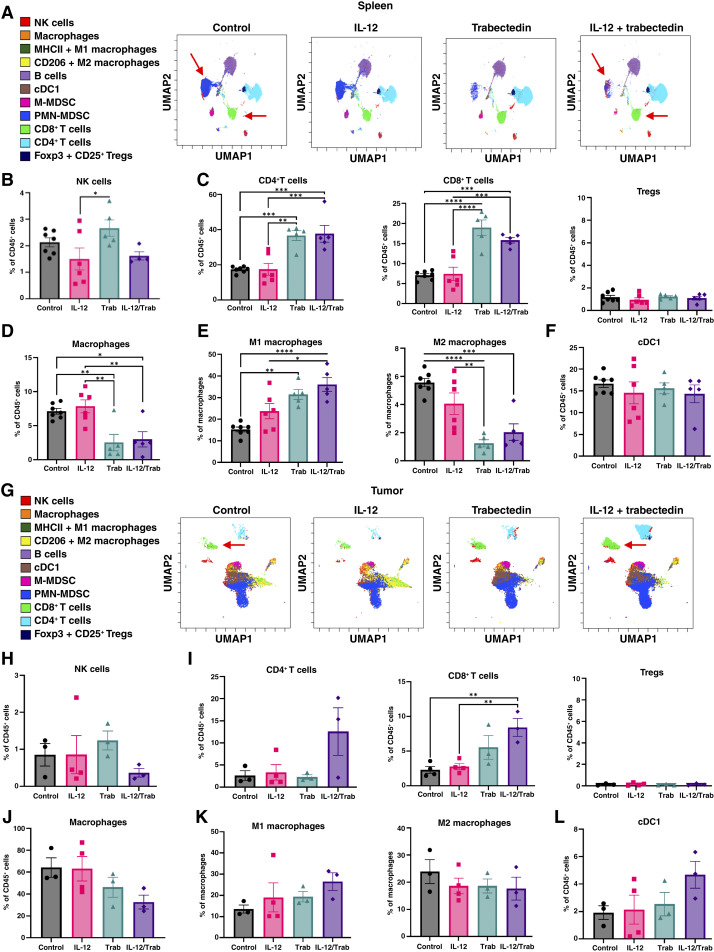
Combination IL-12 and trabectedin selectively reduces immunosuppressive myeloid cells while increasing intratumoral CD8^+^ T cells. Mass cytometry analysis of splenic and tumor-infiltrating immune cells from 4T1 tumor–bearing mice treated with PBS control (*n* = 7), IL-12 (*n* = 6), trabectedin (*n* = 6), and IL-12 plus trabectedin (*n* = 4) for 15 days. **A,** Representative Uniform Manifold Approximation and Projection (UMAP) plots of immune cell populations at day 15 in each treatment group clustered in an unsupervised manner from live/CD45^+^ splenocytes. Percentages of (**B**) NK cells and (**C**) CD4^+^ cells, CD8^+^ cells, and Tregs of total live/CD45^+^ splenocytes. **D,** Percentage of total macrophages of live/CD45^+^ splenocytes and (**E**) the proportions of M1-like and M2-like macrophages of total macrophages. **F,** Percentage of cDC1 cells of live/CD45^+^ splenocytes. **G,** Representative UMAP plots of tumor-infiltrating immune cells from mice treated with PBS control (*n* = 3), IL-12 (*n* = 4), trabectedin (*n* = 3), and IL-12 plus trabectedin (*n* = 3) clustered in an unsupervised manner from live/CD45^+^ cells. **H,** Percentages of NK cells and (**I**) CD4^+^ cells, CD8^+^ cells, and Tregs of live/CD45^+^ tumor–infiltrating cells. **J,** Percentage of total macrophages of live/CD45^+^ tumor-infiltrating cells and (**K**) proportion of M1 and M2 macrophages of total macrophages. **L,** Percentage of cDC1 cells of live/CD45^+^ tumor-infiltrating cells. An ordinary one-way ANOVA with the Tukey multiple comparisons test was used for statistical analysis of bar graphs. Data represent mean ± SEM. *, *P* < 0.05; **, *P* < 0.01; ***, *P* < 0.001; ****, *P* < 0.0001. Trab, trabectedin.

To further investigate the immunologic effects of IL-12 and trabectedin therapy, mass cytometry was performed on the spleens and tumors of treated mice. A 30-antibody panel was developed to allow for the evaluation of multiple immune populations, phenotypes, and activation states (Supplementary Tables S1 and S2). Representative Uniform Manifold Approximation and Projection plots from spleens of each treatment group are presented in Fig. 3A. These illustrate the overall decrease in MDSC and increase in T cells after trabectedin and combination IL-12 and trabectedin therapies. Trabectedin alone also induced an increase in splenic NK cells over IL-12 alone (*P* < 0.05; Fig. 3B). Trabectedin (alone or with IL-12) led to significant increases in splenic CD4^+^ and CD8^+^ T-cell levels (both *P* < 0.002 vs. control and IL-12) without increasing regulatory T cells (Treg; Fig. 3C). Coinciding with the increase in lymphocytes, the overall macrophage levels were significantly reduced in spleens from trabectedin- and combination-treated mice (both *P* < 0.05 vs. control and IL-12; Fig. 3D). Notably, the proportion of MHCII^hi^ M1-like macrophages was significantly increased in both these groups (both *P* < 0.002 vs. control), whereas the proportion of CD206^hi^ M2-like macrophages was significantly decreased (both *P* < 0.001 vs. control; Fig. 3E). These results suggest a selective reduction in immunosuppressive myeloid cells rather than pan-myelosuppression. Other populations such as conventional type 1 DCs (cDC1), myeloid DCs, plasmacytoid DCs, and B cells were not significantly changed with IL-12 and/or trabectedin treatment (Fig. 3F; Supplementary Fig. S3A and S3B).

Mass cytometry was also performed on tumors from these mice, and the representative Uniform Manifold Approximation and Projection plots are presented in Fig. 3G. PMN-MDSC, M-MDSC (Supplementary Fig. S3C), NK-cell (Fig. 3H), CD4^+^ T-cell, Treg (Fig. 3I), and macrophage (total, M1-like, or M2-like; Fig. 3J and K) levels in the tumor did not significantly change with IL-12 and/or trabectedin treatment, which is in contrast to the splenic results. However, the combination of IL-12 and trabectedin led to a significant increase in intratumoral CD8^+^ T cells (*P* = 0.006 vs. control, *P* = 0.01 vs. IL-12; Fig. 3I). The CD8^+^ T cells present in combination-treated tumors also exhibited a significantly higher proportion of naïve (CD62L^+^CD44^−^) cells than any other treatment group (*P* = 0.04 vs. trabectedin, *P* = 0.05 vs. IL-12, *P* = 0.07 vs. control; Supplementary Fig. S3D), possibly indicative of a developing anticancer immune response (31). This was the only population significantly affected by combination therapy as myeloid dendritic cells, plasmacytoid dendritic cells, and B cells remained essentially unchanged (Supplementary Fig. S3E and S3F). There was an increase in cDC1s in combination-treated tumors, although this did not reach significance (*P* = 0.25 vs. control; Fig. 3L). These results indicated that combination IL-12 and trabectedin led to a unique increase in intratumoral CD8^+^ T cells.

### Digital spatial profiling suggests increased immune cell activation in combination-treated tumors

**Figure 4.**
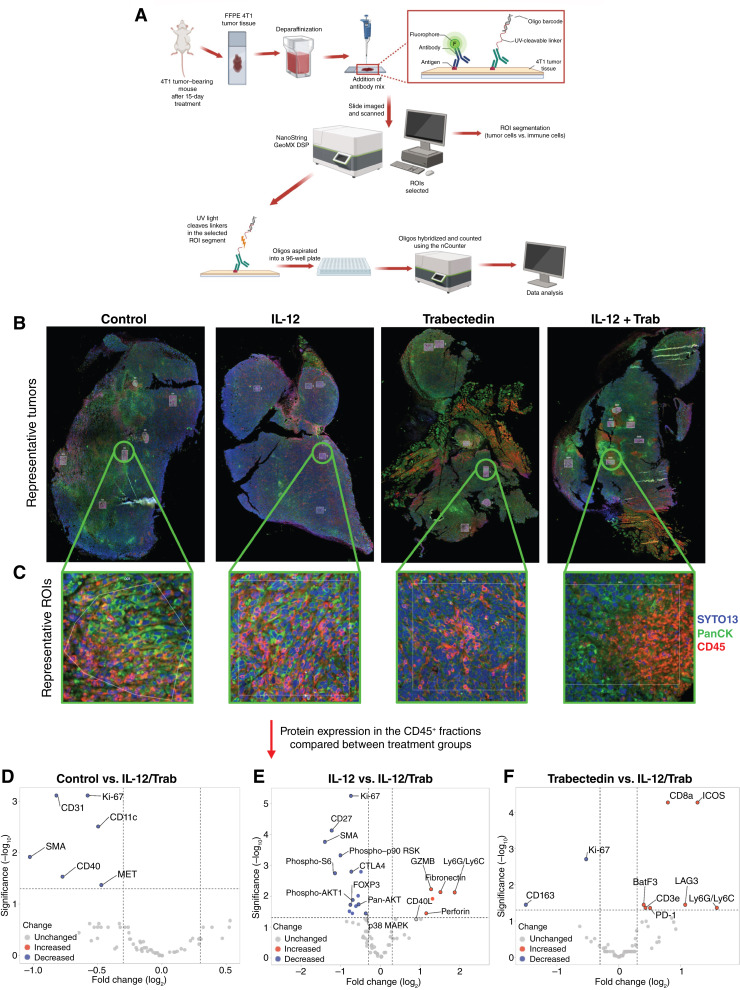
Digital spatial profiling reveals areas of enhanced and activated immune cell infiltration in combination-treated tumors. **A,** Schematic detailing the digital spatial profiling workflow. 4T1 tumors were harvested and mice treated for 15 days with PBS control, IL-12, trabectedin, or IL-12 plus trabectedin and sectioned into formalin-fixed paraffin-embedded (FFPE) tissue slides. **B,** Representative images of tumor slides immunofluorescently stained with an antibody mix for nuclei (SYTO13, blue), immune cells (CD45, red), and tumor cells (PanCK, green). Rectangular boxes represent areas selected as ROIs. **C,** Representative ROIs chosen from each treatment group magnified at 20×. Volcano plots of differentially expressed proteins in CD45^+^ fractions of ROIs between (**D**) control-treated and IL-12 plus trabectedin (IL-12/Trab)–treated tumors, (**E**) IL-12–treated and IL-12/Trab–treated tumors, and (**F**) trabectedin-treated and IL-12/Trab–treated tumors. Red colored points indicate a protein with higher expression in IL-12/Trab–treated tumors compared with control or single agent–treated tumors. Statistical analysis of differential protein expression between treatment groups was determined using a linear mixed model followed by *P* value correction using the Benjamini–Hochberg procedure. Adjusted *P* values < 0.05 were considered significant. Trab, trabectedin.

Digital spatial profiling was used to evaluate protein-level changes in the immune compartment of tumors from 4T1 tumor–bearing mice treated with control PBS, IL-12, trabectedin, or IL-12 plus trabectedin. A schematic detailing of the experimental workflow is shown in Fig. 4A. Representative fluorescently stained tumor images are shown in Fig. 4B and highlight the CD45^+^ immune cell (red), PanCK^+^ tumor cell (green), and SYTO13 nuclei staining (blue). Images are representative of one of three tumors analyzed per treatment group. Select ROIs are contained within the distinct boxes shown in Fig. 4B and identify areas chosen for further investigational profiling. Combination-treated tumors consistently displayed areas of high-density immune cell staining in close proximity to tumor cells, which was not seen in any other treatment group. Several ROIs were chosen in these areas to better understand the immunologic responses occurring. A representative ROI from each treatment group is shown in Fig. 2C at 20× magnification to highlight the distinct patterns in immune and tumor cell staining. Each ROI was segmented into CD45^+^ immune fractions and PanCK^+^ tumor fractions by manually selecting CD45 and PanCK fluorescence thresholds that visually maximized the individual cell types, allowing for the evaluation of 71 protein targets expressed within each individual fraction (targets are listed in Supplementary Table S3). A total of 12 ROIs were selected from each tumor, resulting in a total of 36 ROIs per treatment group used in the final analysis. Protein expression was quantitated within immune fractions of each ROI, and differential expression analysis was performed among the four treatment groups. Within the immune compartment, there were 6 differentially expressed proteins between control- and combination-treated tumors, 21 differentially expressed proteins between IL-12–treated and combination-treated tumors, and 9 differentially expressed proteins between trabectedin- and combination-treated tumors (Fig. 4D–F; Supplementary Table S4A–S4C). Levels of Ki-67 (*P* < 0.001), CD11c (*P* < 0.01), CD40 (*P* = 0.03), CD31 (*P* < 0.001), SMA (*P* = 0.01), and MET (*P* = 0.04) were significantly lower in the CD45^+^ fractions of combination-treated tumors compared with control-treated tumors (Fig. 4D; Supplementary Table S4A). Compared with IL-12–treated tumors, combination-treated tumors had significantly lower levels of several proteins in the PI3K/AKT/mTOR and MAPK signaling pathways (all *P* < 0.05), which could be due to the influence of tumor cells in the vicinity. Combination-treated tumors also had significantly lower levels of CTLA-4 (*P* = 0.002), an inhibitory molecule for DC–T-cell interactions compared with IL-12–treated tumors (Fig. 4E; Supplementary Table S4B; ref. 32). Combination-treated tumors had significantly higher levels of NK/T-cell cytotoxicity markers granzyme B and perforin (both *P* < 0.04) and increased co-stimulatory molecule CD40L (*P* = 0.056) compared with IL-12–treated tumors, suggesting increased intratumoral immune cell activation when trabectedin was added to IL-12 (Fig. 4E; Supplementary Table S4B). Finally, compared with trabectedin alone, combination-treated tumors had significantly higher levels of the T-cell markers CD3e and CD8a (*P* = 0.04 and *P* < 0.0001, respectively) and checkpoint molecules PD-1 and LAG3 (both *P* < 0.04), the latter of which is associated with improved overall and relapse-free survival in TNBC (33). The cDC1-required transcription factor BATF3 and the co-stimulatory molecule implicated in T-cell activation and differentiation ICOS were also significantly elevated (both *P* < 0.5; Fig. 4F; Supplementary Table S4C; refs. 34, 35). A heatmap showing the expression levels of all 71 proteins is shown in Supplementary Fig. S4. In all four treatment groups, there was low expression of the B-cell marker CD19 and CD4^+^ T-cell marker/Treg markers CD4 and FOXP3, mirroring what was seen by intratumoral mass cytometry (Supplementary Fig. S4).

### NK-cell depletion reduces combination efficacy, chemokine secretion, and intratumoral CD8^+^ T cells

**Figure 5.**
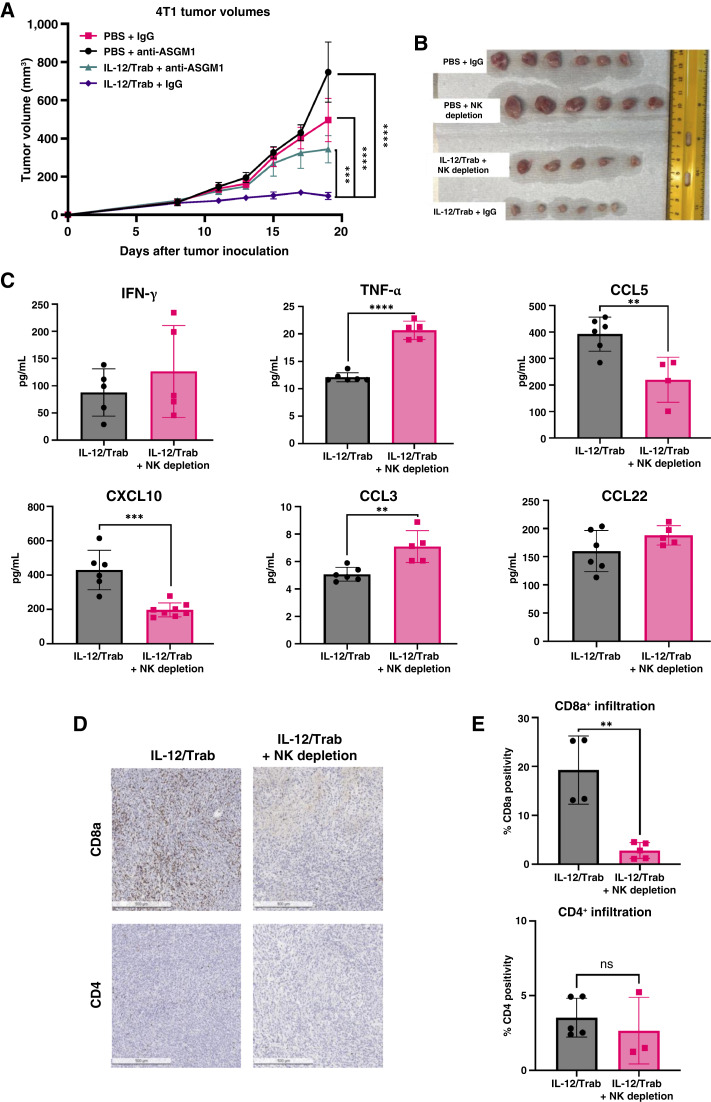
NK-cell depletion reduces combination-induced efficacy and reduces intratumoral CD8a. BALB/c mice were inoculated with 4T1 cells and divided into one of two groups once tumors reached ∼50 mm^3^. Mice were either depleted of NK cells using anti–asialo-GM1 antibody or treated with an IgG isotype control antibody. Anti–asialo-GM1 was administered by intraperitoneal injection at 100 μg/100 μL 3 days prior to the start of IL-12 and trabectedin treatment and every 4 days thereafter. The IgG isotype control antibody was administered in nondepleted mice at the same time points and dose as the depletion antibody. NK-depleted mice were treated with PBS control (*n* = 6) or intraperitoneal injection of 0.5 μg IL-12 3×/week and intravenous injection of 0.15 mg/kg trabectedin 1×/week (*n* = 5). Nondepleted mice were similarly treated with either PBS control (*n* = 8) or IL-12 plus trabectedin (*n* = 6). **A,** Tumor growth curves throughout the 12-day treatment. **B,** Images of tumors after excision. For statistical analyses of tumor volumes, linear mixed modeling was used to model the longitudinal tumor volume for mice under each treatment. Comparisons were done at each time point and averaged across all time points using t-statistics. The Tukey–Kramer method was used for adjusting raw *P* values for multiple comparisons across treatment groups. Values are the mean ± SEM of tumor volumes at each time point. **C,** Plasma cytokine and chemokine levels in mice at day 19. Values represent individual samples and are shown as ± SEM. **D,** Representative images (5× magnification) of IHC staining for CD8a and CD4 performed on 4T1 tumors from mice treated with IL-12 and trabectedin ± NK-cell depletion. **E,** Quantification of IHC staining as reported by percent CD8a (*n* = 4–5) and percent CD4 (*n* = 3–5) positivity/slide. Values represent individual tumor slides and are shown as mean ± SEM. Statistical analyses of bar graphs were performed using two-tailed unpaired student *t* tests. **, *P* < 0.01; ***, *P* < 0.001; ****, *P* < 0.0001. Trab, trabectedin.

Based on the role of NK cells in IL-12 therapy and the increased cytotoxic/activation markers detected by digital spatial profiling, we investigated how NK cells may be contributing to the efficacy of IL-12 and trabectedin therapy. To test this, antibody-mediated NK-cell depletion was performed using anti–asialo-GM1 prior to IL-12 and trabectedin administration. The efficiency of NK-cell depletion was >85% as confirmed by flow cytometric analysis of splenocytes (Supplementary Fig. S5A). No significant differences were detected in CD3^+^ T cells following administration of anti–asialo-GM1 (Supplementary Fig. S5B). NK-cell depletion significantly reduced the efficacy of combination therapy, as evidenced by the significant increase in tumor growth and final volumes (*P* < 0.001; Fig. 5A and B; Supplementary Fig. S6). This result suggested an essential role of NK cells in the efficacy of IL-12 and trabectedin therapy. NK-cell depletion affected downstream immune responses induced by combination therapy, as evidenced by alterations in cytokine and chemokine levels (Fig. 5C). Circulating levels of CCL5, an NK cell–derived chemokine, were significantly reduced with NK-cell depletion (*P* < 0.01; Fig. 5C). Circulating levels of the DC-derived chemokine CXCL10 were also significantly reduced with NK-cell depletion (*P* < 0.01; Fig. 5C). In contrast, levels of TNF-α and CCL3 were significantly increased in the context of NK-cell depletion (both *P* < 0.01; Fig. 5C). Levels of GM-CSF, granzyme B, CCL2, and CCL4 were below the limit of detection at this time point in both treatment groups, and IFN-γ and CCL22 levels were not significantly altered upon NK-cell depletion. Based on the previously described roles of NK cell–derived CCL5 and DC-derived CXCL10 in recruiting immune cells, we investigated whether NK-cell depletion might affect the levels of intratumoral CD8^+^ T cells in combination-treated mice (34). CD8a^+^ cells were significantly reduced in the tumors of combination-treated mice that had been depleted of NK cells (*P* = 0.001; Fig. 5D and E). On the other hand, the levels of CD4^+^ cells did not change with NK-cell depletion, again suggesting a specific effect on intratumoral CD8^+^ T cells (Fig. 5D and E).

### CD8^+^ T-cell depletion also reduces combination-induced efficacy

**Figure 6.**
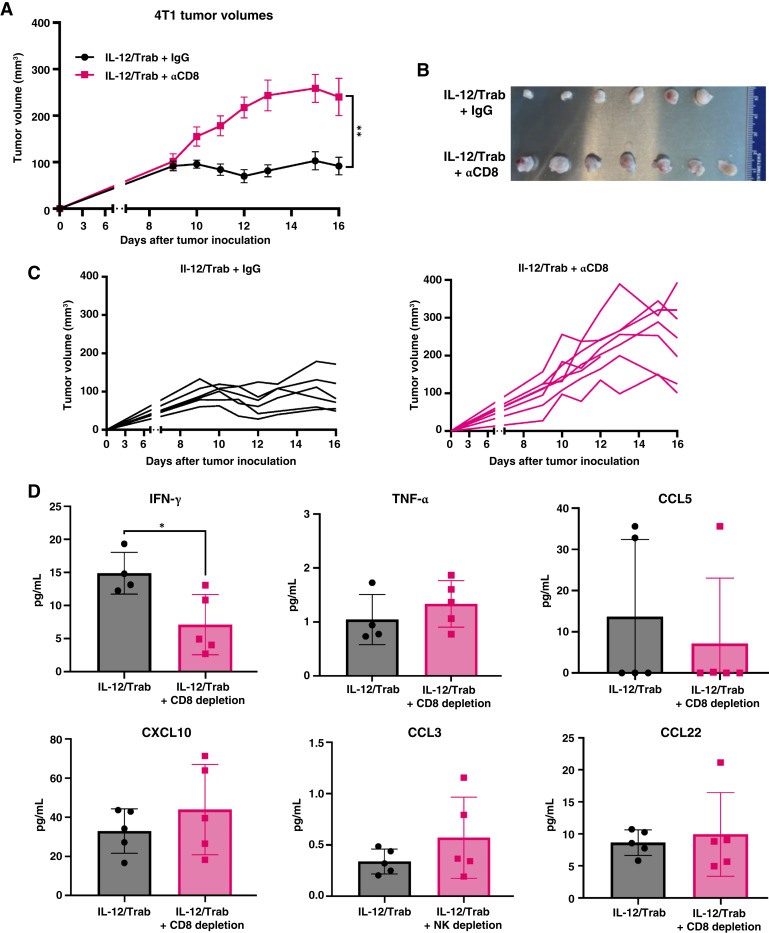
CD8a depletion abrogates IL-12/trabectedin-induced tumor control. BALB/c mice were inoculated with 4T1 cells. Once tumors reached ∼50 mm^3^, mice were either depleted of CD8a^+^ cells using α-CD8a antibody (*n* = 7) or treated with an IgG isotype control antibody (*n* = 6). CD8a^+^ depletion was performed through intraperitoneal injection of an αCD8 antibody 1 day prior to the start of IL-12 and trabectedin treatment (at a dose of 200 μg) and every 4 days thereafter (at a dose of 100 μg). Nondepleted mice were treated with the same dose of control antibody at the same time points. Both groups were treated with 0.5 μg IL-12 i.p. 3 ×/week and 0.15 mg/kg trabectedin i.v. 1×/week. **A,** Tumor growth curves throughout the 8-day treatment. **B,** Tumor images after excision. **C,** Individual tumor growth curves for IL-12 and trabectedin + IgG–treated mice and IL-12 and trabectedin + αCD8–treated mice. **D,** Plasma cytokine and chemokine levels in mice (*n* = 4–6) at day 16. For statistical analyses of tumor volumes, linear mixed modeling was used to model longitudinal tumor volume for mice under each treatment. Comparisons were done at each time point and averaged across all time points using t-statistics. The Tukey–Kramer method was used for adjusting raw *P* values for multiple comparisons across treatment groups. Statistical analyses of bar graphs were performed using two-tailed unpaired student *t* tests. Data represent mean ± SEM. *, *P* < 0.05; **, *P* < 0.01. Trab, trabectedin.

To determine if CD8^+^ T cells played a role in the efficacy of combination therapy, a CD8 depletion study was performed. Mice were treated with the combination of IL-12 and trabectedin following CD8^+^ T-cell depletion or control treatment. The efficiency of CD8^+^ T-cell depletion was >99% (Supplementary Fig. S7). The efficacy of combination therapy was significantly reduced in the context of CD8 T-cell depletion (*P* = 0.002; Fig. 6A). Tumor growth and overall volumes were significantly higher in mice depleted of CD8^+^ T cells prior to treatment (Fig. 6B and C). Thus, the efficacy of IL-12 and trabectedin therapy seems to rely on both the CD8^+^ T-cell and NK-cell compartments. CD8^+^ T-cell depletion significantly reduced levels of IFN-γ induced by combination therapy, unlike what was seen with NK-cell depletion (*P* = 0.02; Fig. 6D). Levels of TNF-α and chemokines did not change with CD8 T-cell depletion (Fig. 6D).

### Combination IL-12 and trabectedin therapy enhances immune checkpoint blockade

**Figure 7.**
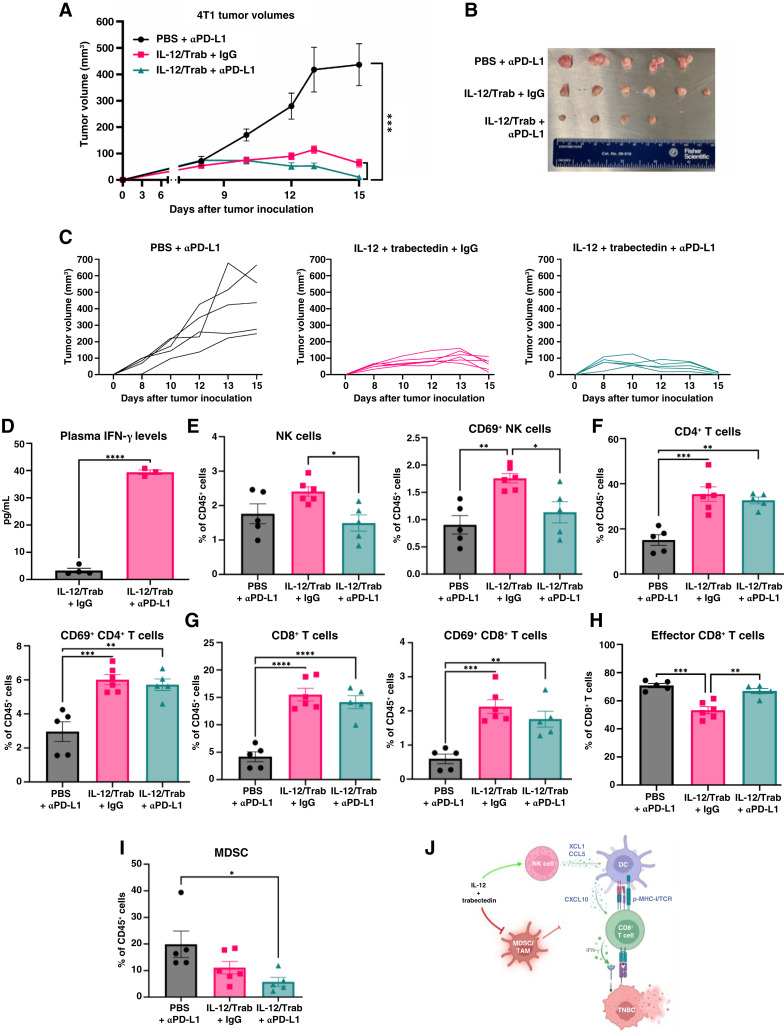
Anti–PD-L1 therapy improves response to combination IL-12 and trabectedin treatment. Mice bearing ∼50 mm^3^ 4T1 tumors were treated with 0.5 μg IL-12 i.p. 3×/week and 0.15 mg/kg trabectedin i.v. 1×/week for 7 days with either αPD-L1 therapy (100 μg 3×/week, *n* = 5) or isotype control IgG (100 μg 3×/week, *n* = 6). **A,** Tumor growth curves throughout treatment. **B,** Tumor images after excision. One mouse from the triple combination group had a complete response and therefore had no tumor for imaging. For statistical analyses of tumor volumes, linear mixed modeling was used to model longitudinal tumor volume for mice under each treatment. Comparisons were done at each time point and averaged across all time points using t-statistics. The Tukey–Kramer method was used for adjusting raw *P* values for multiple comparisons across treatment groups. **C,** Individual tumor growth curves of mice treated with PBS + αPD-L1, IL-12 and trabectedin + IgG, and IL-12 and trabectedin + αPD-L1. **D,** Plasma levels of IFN-γ in mice treated with IL-12 and trabectedin + IgG or αPD-L1 at day 15 (*n* = 3–5). Statistical analysis was performed using two-tailed unpaired student *t* tests. **E–I,** Percentages of splenic immune cells at day 15. An ordinary one-way ANOVA with the Tukey multiple comparisons test was used for statistical analyses of mass cytometry data. Data represent mean ± SEM. **J,** Proposed mechanism of IL-12 and trabectedin–induced antitumor immunity. Trabectedin reduced inhibitory immune cells (MDSC and TAMs) in mice bearing TNBC tumors. Without the presence of a highly immunosuppressive TME, trabectedin and/or IL-12 may be able to activate NK cells and lead to increased CCL5 and XCL1 production. CCL5 and XCL1 can attract CXCL10-producing cDC1 into the TME, where these cells can cross-present antigen to CD8^+^ T cells and promote an improved antitumor response. *, *P* < 0.05; **, *P* < 0.01; ***, *P* < 0.001; ****, *P* < 0.0001. TCR, T-cell receptor; Trab, trabectedin.

After confirming that CD8^+^ T cells played a significant role in combination efficacy, we sought to determine whether treatment with IL-12 and trabectedin could further enhance the immune response to an inhibitor of the PD-1/PD-L1 immune checkpoint. Mice bearing 4T1 tumors were treated with either an anti–PD-L1, IL-12 plus trabectedin, or IL-12 plus trabectedin with an anti–PD-L1. Single-agent anti–PD-L1 had little effect on tumor growth, whereas treatment with IL-12 and trabectedin led to the expected significant decrease in tumor growth (*P* = 0.002; Fig. 7A and B). However, the combination of IL-12 and trabectedin with an anti–PD-L1 significantly reduced tumor growth and volumes beyond that achieved with IL-12 and trabectedin therapy alone and led to one complete response (*P* = 0.03; Fig. 7A–C). The addition of anti–PD-L1 to IL-12 and trabectedin therapy also significantly increased circulating levels of IFN-γ (*P* < 0.0001; Fig. 7D). As the immune-stimulating effects of anti–PD-L1 therapy are seen predominantly in T and NK cells, changes in these populations were assessed after treatment. IL-12 and trabectedin therapy increased the percentages of splenic NK cells, CD4^+^ T cells, and CD8^+^ T cells compared with anti–PD-L1 therapy alone (Fig. 7E–G). Levels of CD69^+^ within NK cells, CD4^+^ cells, and CD8^+^ T cells were also significantly increased with IL-12 and trabectedin therapy compared with anti–PD-L1 (all *P* < 0.01; Fig. 7E–G). The addition of anti–PD-L1 therapy to IL-12 and trabectedin led to decreased levels of total and CD69^+^ NK cells (Fig. 7E) but increased the proportion of CD8^+^ T cells with an effector phenotype (*P* = 0.001; Fig. 7H). This triple combination also significantly decreased the proportion of naïve and central memory CD8^+^ T cells as well as Treg compared with IL-12 and trabectedin alone (all *P* < 0.05; Supplementary Fig. S8A and S8B). Total MDSC levels were further decreased with the addition of anti–PD-L1 therapy to IL-12 and trabectedin, and the proportion of M1-like macrophages was further increased (Fig. 7I; Supplementary Fig. S8C).

## Discussion

Previous work has described the essential role of NK cells in clinical responses to IL-12 therapy (18). However, immunosuppressive cells such as MDSC can inhibit IL-12–induced NK-cell activation and may therefore represent a barrier to beneficial responses (14). The present study utilized trabectedin as an immunosuppressive myeloid cell–depleting agent to investigate whether removing this barrier to response prior to IL-12 administration could improve its antitumor activity. TNBC was chosen as the setting to test this combination because of the high MDSC levels, presence of tumor-induced NK-cell dysfunction, and lack of targeted treatment options (15, 16). In this study, the addition of trabectedin to IL-12 therapy led to significantly higher tumor regression in both 4T1 and EMT6 models of TNBC than single-agent controls. Significant reductions in splenic MDSC and M2-like but not M1-like macrophages after treatment supported the ability of trabectedin to reduce suppressive myeloid cells in this model. The ability of this combination to sensitize 4T1 tumors to anti–PD-L1 therapy also signifies its potential application to immune-based treatments, in general. As a single agent, trabectedin led to a significant increase in CD69 expression and T-BET (*Tbx21*), TRAIL (*TNFSF10*), Fas ligand (*FASLG*), and lymphotactin (*XCL1*) mRNA expression in NK cells. In the presence of human TNBC cells, IL-12 and trabectedin significantly increased NK-cell cytotoxicity and activation and secretion of IFN-γ, TNF-α, and granzyme B. Collectively, these results indicate an antitumor effect of trabectedin when combined with IL-12 in TNBC.

Mechanistically, this study suggests that trabectedin and IL-12 therapy may stimulate NK cells *in vivo* to secrete immune-mediating chemokines CCL5 and XCL1. These factors can attract CXCL10-producing cDC1 into the TME, ultimately leading to increased intratumoral CD8^+^ T cells and improved antitumor responses (Fig. 7J). This hypothesis is supported by the decrease in trabectedin and IL-12 antitumor efficacy when NK cells were depleted. The concurrent significant decrease in CCL5 and CXCL10 following NK-cell depletion suggests that trabectedin and IL-12 may be stimulating NK-cell induction of these immune-mediating chemokines. NK cells have been shown to be the main producers of intratumoral CCL5 and XCL1 in solid tumors (34). Of note, the *in vitro* treatment of NK cells with trabectedin significantly increased *XCL1* mRNA expression. XCL1 and CCL5 are crucial chemokines in antitumor immunity because of their ability to recruit XCR1^+^ cDC1s into the TME (36). Intratumoral accumulation of cDC1s has been shown to be dependent on NK cell–derived CCL5, and XCL1 and cDC1 have also been shown to be one of the main producers of the T cell–attracting chemokine CXCL10 (34). The ability of cDC1 to cross-present antigens to CD8^+^ T cells within the TME uniquely enables them to elicit antitumor immune responses (36). In the present model, combination-treated tumors had increased levels of cDC1 and CD8^+^ T cells. The preponderance of naïve-like CD8^+^ T cells suggests that this DC–T-cell interplay may play a part in mediating the IL-12 and trabectedin–induced antitumor immune response. The high expression of the cDC1-required transcription factors BATF3 and CD8a in combination-treated tumors also supports the involvement of cDC1s.

Additional studies exploring the interplay among NK cells, cDC1, and CD8^+^ T cells suggest that this axis may contribute to antitumor efficacy (34). Böttcher and colleagues (34) analyzed The Cancer Genome Atlas data and found that a cDC1 signature was positively correlated with an XCL1/XCL2 and CCL5 chemokine signature and an NK-cell signature in breast cancer, melanoma, and lung adenocarcinoma. When analyzing TNBC samples, this group found a significant positive association between cDC1 and NK cell signature genes and patient survival. This suggests that this NK/cDC1/T-cell axis may contribute to antitumor efficacy in TNBC. Image-based deep learning has also shown intratumoral clustering of cDC1, and CD8^+^ T cells act as a feature of protective anticancer immunity through the intratumoral cross-presentation of tumor antigens and subsequent CD8^+^ T-cell differentiation and expansion (37). Several groups have found that intratumoral cDC1 are associated with increased CD8^+^ T cells in the TME and increased immunotherapeutic success (38, 39). This is congruent with our finding that IL-12 and trabectedin could sensitize 4T1 tumors to anti–PD-L1 therapy and that this triple combination could induce higher tumor regression than IL-12 and trabectedin alone. The finding that NK-cell depletion also significantly reduced intratumoral CD8^+^ T cells supports this notion. However, follow-up studies will be needed to confirm the reliance of IL-12 and trabectedin therapy on an NK/cDC1/T-cell axis.

The preclinical efficacy of trabectedin has been shown in several tumor models, such as sarcoma and multiple myeloma (23, 28). As in our model, trabectedin was found to deplete MDSC and M2-like but not M1-like macrophages in a murine model of Ewing sarcoma (23). The combination of trabectedin and oncolytic herpes simplex virus also led to tumor regression in Ewing sarcoma, suggesting that the addition of an immune-stimulating agent to trabectedin may be an effective therapeutic strategy in solid tumors (23). In an immunocompetent model of osteosarcoma, the combination of trabectedin and oncolytic herpes simplex virus was also effective, with antitumor efficacy being mediated by NK and CD8^+^ T cells (40). Single-agent trabectedin induced mRNA expression of *IFNG* and *GZMB* in CD8^+^ T cells from patients with chronic lymphocytic leukemia treated *in vitro*, which aligns with our findings in NK cells (41). Additionally, the findings by Cucè and colleagues (28) and Allavena and colleagues (21) that trabectedin leads to increased tumor cell NKG2D ligand expression and inhibits transcription of cytokines linked to NK-cell dysfunction, such as IL-6, suggests that trabectedin may indirectly activate NK cells in the setting of cancer. The ability of trabectedin to induce NK-cell activation and transcription of T-BET, IFN-γ, and granzyme B suggests a potential direct mechanism of NK-cell stimulation that bears further investigation. However, phospho-STAT4 levels were similar between IL-12–treated and combination-treated NK cells, suggesting that trabectedin was not exerting an effect on this IL-12 signaling pathway. Thus, the pathway through which trabectedin is acting on NK cells remains to be elucidated.

The observation that trabectedin binds to DNA and prevents effective DNA repair led to the finding that tumors lacking functional BRCA2 were hypersensitive to trabectedin-induced apoptosis. This resulted in several clinical trials investigating trabectedin for the treatment of breast cancer (42, 43). A phase II study of trabectedin in patients with advanced, pretreated breast cancer having received at least one regimen of taxanes or anthracyclines resulted in an overall response rate of 14% with 3 of 21 patients achieving a partial response (PR; ref. 42). Another phase II study of trabectedin monotherapy was conducted in pretreated patients with metastatic breast cancer harboring a germline mutation in *BRCA1/2* (43). Clinical responses were higher in this patient population with six PRs. Trabectedin monotherapy in the TNBC cohort led to unconfirmed PRs in 2 of 43 patients (44). The existence of high levels of chemotherapeutic resistance in the triple-negative subtype could represent a reason for the reduced effectiveness of trabectedin monotherapy in this setting (10). The addition of trabectedin to an immune-based therapy may represent a promising new treatment strategy in this patient population.

Other studies have suggested that combining trabectedin with immune-stimulating agents may improve antitumor efficacy. For example, a preclinical study found pretreatment with trabectedin improved the response to anti–PD-1 in a murine model of fibrosarcoma and led to upregulation of *CD8*, *GZMB*, *PRF1*, and *CXCL10* mRNA expression in trabectedin-treated tumors (45). Several clinical trials have also tested trabectedin with various immune checkpoint inhibitors such as durvalumab, nivolumab, ipilimumab, and nivolumab plus ipilimumab (46–49). The best overall response rate was 25.3% in a study that evaluated trabectedin with nivolumab plus ipilimumab as a first-line treatment for advanced soft-tissue sarcoma (49). These results suggest that trabectedin could play a role as an adjunct to immunologic therapy in specific settings. Investigating the efficacy of IL-12 and trabectedin in other solid tumor types is an important future direction of this study. However, thorough monitoring of adverse events when combining two immune-stimulating agents in patients is imperative. Investigating whether an intratumoral dose of IL-12 could provide the same antitumor effect with less side effects is an important future direction. Although pretreatment with corticosteroids has been shown to reduce trabectedin-induced hepatotoxicity and myelosuppression, this approach could inhibit IL-12–induced immune responses (50).

In summary, this study provides preclinical evidence for the efficacy of IL-12 and trabectedin therapy in the treatment of TNBC. The *in vitro* findings suggest that there may be a previously undescribed role for trabectedin in the activation of NK cells and potential for increased activation when combined with IL-12. The finding that this combination further sensitized 4T1 tumors to anti–PD-L1 therapy supports additional studies combining IL-12 and trabectedin therapy with immune checkpoint inhibitors.

## Supplementary Material

Supplementary Figure S1 Supplementary Figure S1

Supplementary Figure S2 Supplementary Figure S2

Supplementary Figure S3 Supplementary Figure S3

Supplementary Figure S4 Supplementary Figure S4

Supplementary Figure S5 Supplementary Figure S5

Supplementary Figure S6 Supplementary Figure S6

Supplementary Figure S7 Supplementary Figure S7

Supplementary Figure S8 Supplementary Figure S8

Supplementary Table S1 Supplementary Table S1

Supplementary Table S2 Supplementary Table S2

Supplementary Table S3 Supplementary Table S3

Supplementary Table S4 Supplementary Table S4
