# Supplementary material for: Trabectedin Enhances the Antitumor Effects of IL-12 in Triple-Negative Breast Cancer
Source: Cancer Immunol Res. 2025 Jan 7;13(4):560–76. doi: 10.1158/2326-6066.CIR-24-0775 (PMC11962391; doi:10.1158/2326-6066.CIR-24-0775)
Supplement: Supplementary Figure S1 [file cir-24-0775_supplementary_figure_s1_supps1.pdf]

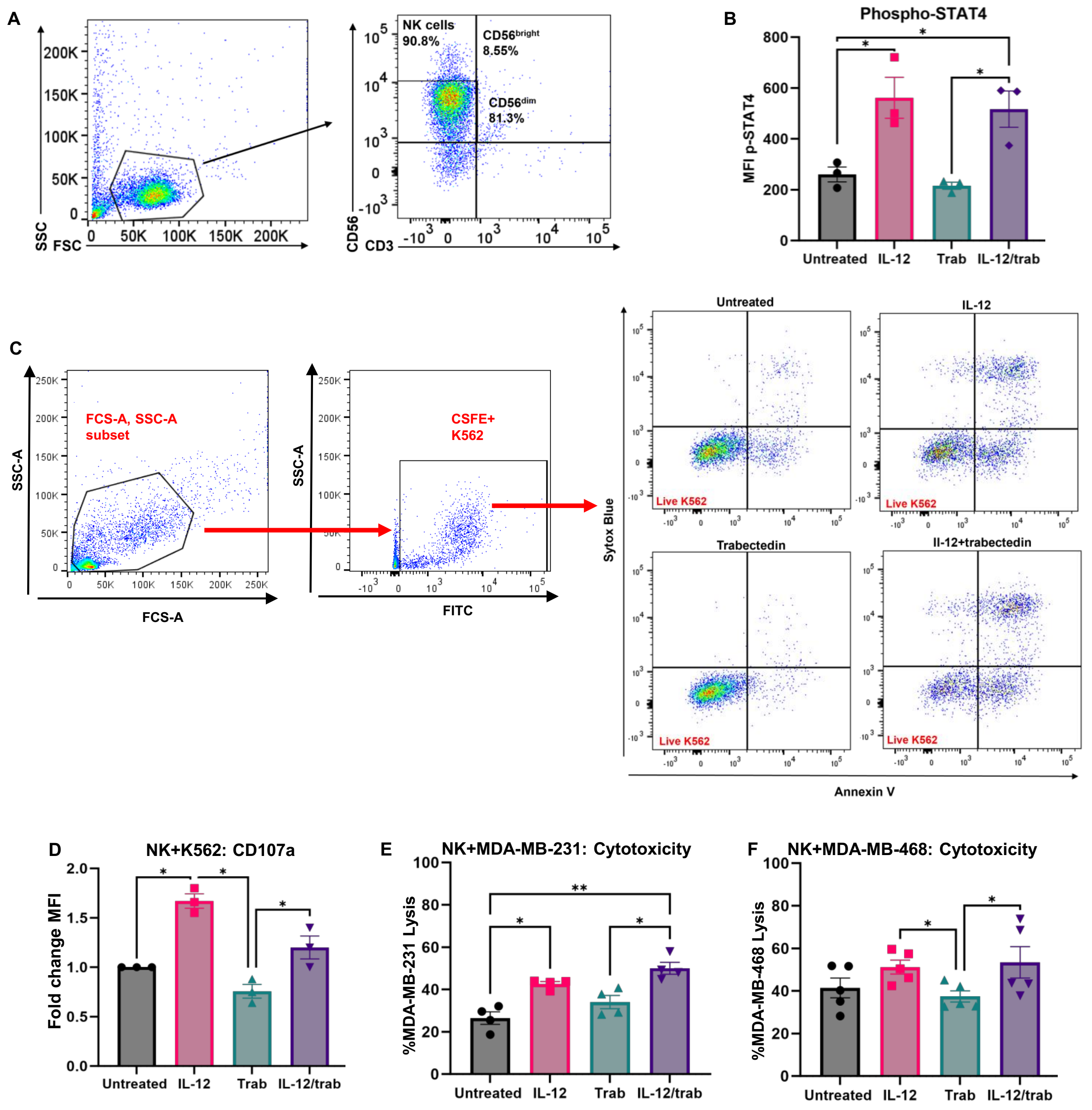

Supplementary Figure S1. Flow cytometry gating strategies and evaluation of NK cell STAT4 phosphorylation and cytotoxicity against tumor cells. (A) Representative flow cytometry gating strategy used to evaluate isolated CD3^{+}CD56^{+} human NK cell purity. (B) Intracellular NK cell phospho-STAT4 staining after 2 h treatment measured by flow cytometry. Values reported are mean fluorescence intensity of phospho-STAT4 staining (MFI) (n=3 donors). (C) Representative experimental gating strategy for NK cell cytotoxicity assays. Plots are representative of n=1 assay against K562 cells. K562 (or human triple-negative breast cancer) tumor cells were gated out using CSFE and cells negative for both Sytox Blue and Annexin V were considered viable. (D) CD107a expression on pre-treated NK cells post-4 h co-culture with K562 cells (n=3). Values reported are fold changes in MFI compared to DMSO treated NK cells. Quantification of tumor cell lysis in an NK cell 4 h cytotoxicity assay against human triple-negative breast cancer cell lines (E) MDA-MB-231 (n=4) and (F) MDA-MB-468 (n=5). Data represent mean ± SEM. Statistical analyses were performed using RM one-way ANOVA with Tukey's multiple comparisons test. *p<0.05, **p<0.01.
