# Supplementary material for: Trabectedin Enhances the Antitumor Effects of IL-12 in Triple-Negative Breast Cancer
Source: Cancer Immunol Res. 2025 Jan 7;13(4):560–76. doi: 10.1158/2326-6066.CIR-24-0775 (PMC11962391; doi:10.1158/2326-6066.CIR-24-0775)
Supplement: Supplementary Figure S2 [file cir-24-0775_supplementary_figure_s2_supps2.pdf]

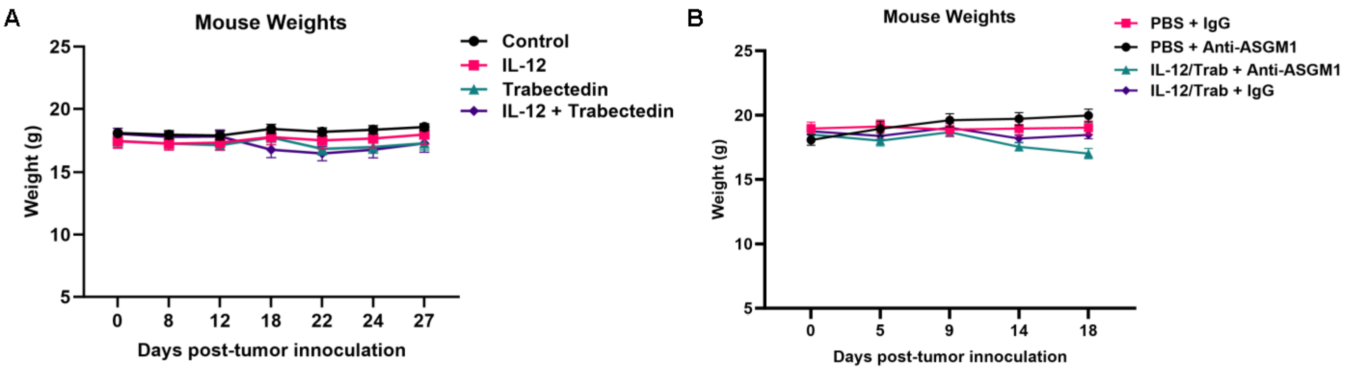

Supplementary Figure S2. The combination of IL-12 and trabectedin therapies did not induce significant weight loss in mice. (A) BALB/c mice weights (g) throughout treatment in 4T1-tumor bearing mice (n=10-12). (B) BALB/c mice weights (g) throughout treatment in 4T1-tumor bearing mice with or without NK cell depletion (n=5-6). For statistical analysis of weight changes, linear mixed modeling was employed to model longitudinal mouse weights under each treatment. Comparisons were done at each time point and averaged across all time points using t-statistics. The Tukey-Kramer method was used for adjusting raw p-values for multiple comparisons across treatment groups. Data represent mean ± SEM.
