# Supplementary material for: Trabectedin Enhances the Antitumor Effects of IL-12 in Triple-Negative Breast Cancer
Source: Cancer Immunol Res. 2025 Jan 7;13(4):560–76. doi: 10.1158/2326-6066.CIR-24-0775 (PMC11962391; doi:10.1158/2326-6066.CIR-24-0775)
Supplement: Supplementary Figure S3 [file cir-24-0775_supplementary_figure_s3_supps3.pdf]

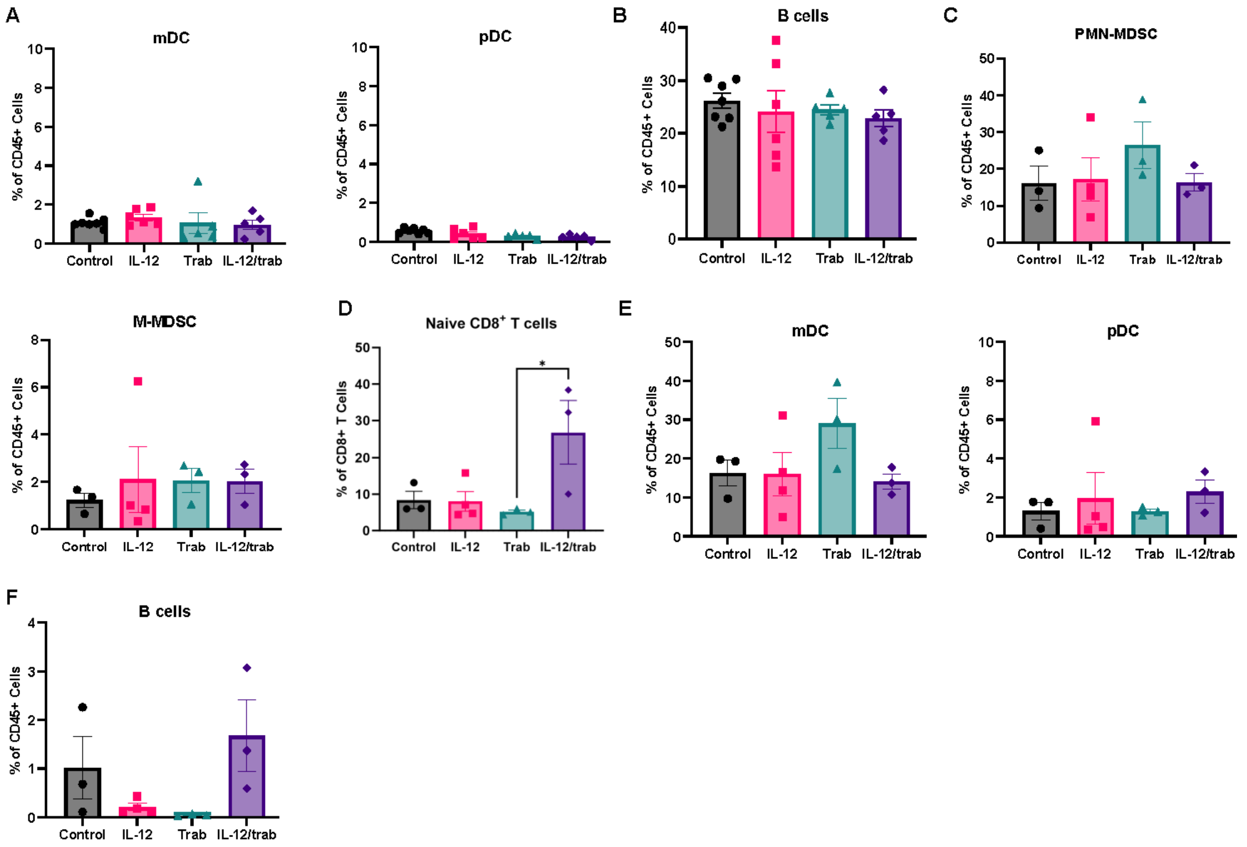

Supplementary Figure S3. Additional splenic and intratumoral immune cell changes following IL-12 and trabectedin treatment in 4T1-tumor bearing mice. (A) Percentages of splenic myeloid (mDC) and plasmacytoid (pDC) dendritic cells and (B) B cells post-15-day treatment (n=5-7). (C) Percentages of intratumoral PMN-MDSC, M-MDSC post-15-day treatment (n=3-4). (D) Percentages of intratumoral naïve ($CD62L^+CD44^-$) $CD8^+$ T cells (E) mDC, pDC and (F) B cells post-15-day-treatment (n=3-4). Statistical analyses were performed using ANOVA with Tukey's multiple comparisons test. Data represent mean ± SEM. *$p<0.05$.
