# Supplementary material for: Trabectedin Enhances the Antitumor Effects of IL-12 in Triple-Negative Breast Cancer
Source: Cancer Immunol Res. 2025 Jan 7;13(4):560–76. doi: 10.1158/2326-6066.CIR-24-0775 (PMC11962391; doi:10.1158/2326-6066.CIR-24-0775)
Supplement: Supplementary Figure S4 [file cir-24-0775_supplementary_figure_s4_supps4.pdf]

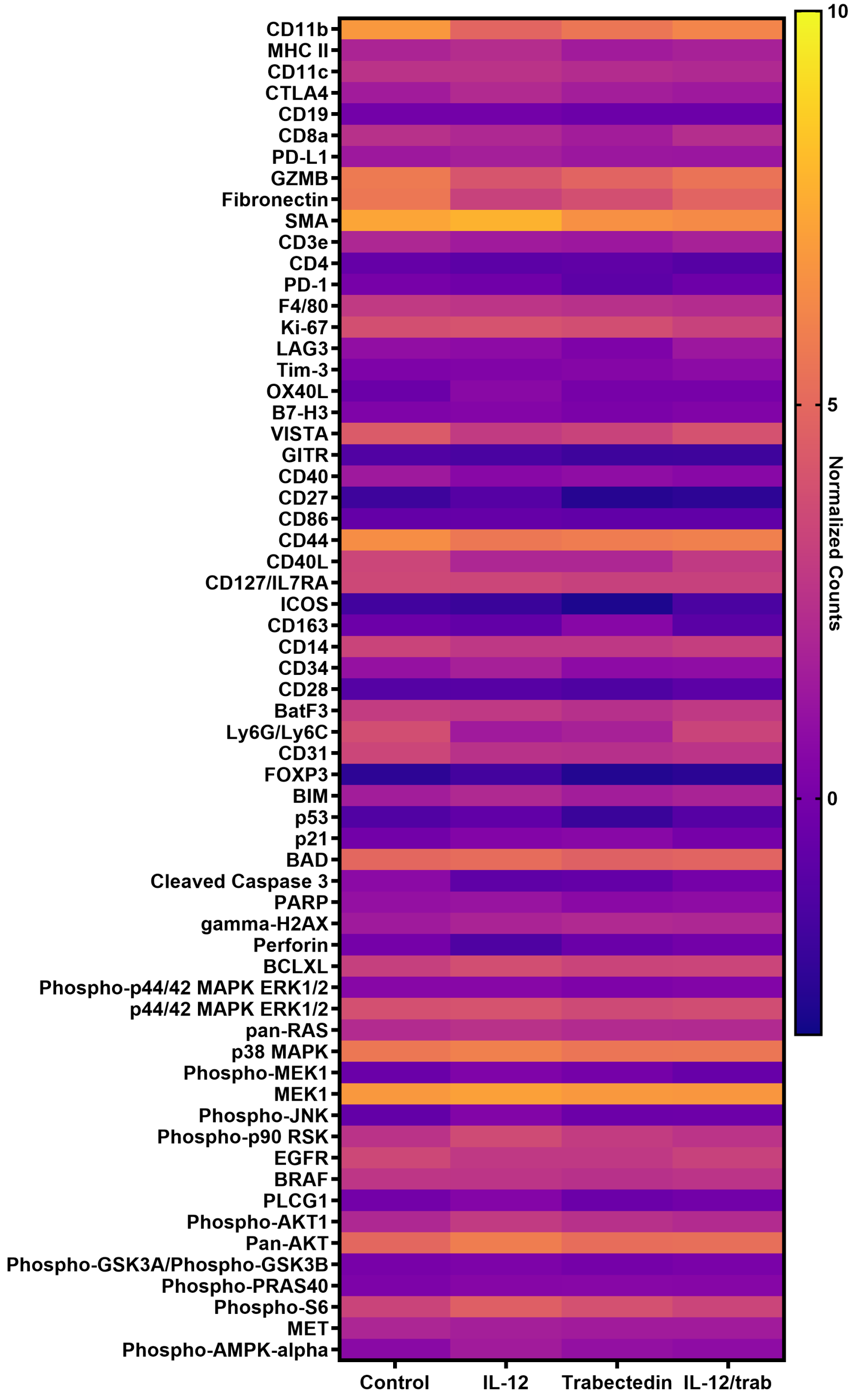

Supplementary Figure S4. Heatmap of protein expression measured via Nanostring digital spatial profiling. Heatmap showing protein expression levels as normalized counts of all proteins assayed within $CD45^+$ ROIs. Yellow color indicates higher counts and purple color indicates lower counts. Data are representative of n=3 tumors per treatment group. Statistical analysis of differential protein expression between treatment groups was determined using a linear mixed model to account for multiple ROI sections within individual samples and batch effects between runs followed by p-value correction using Benjamini–Hochberg (BH) procedure.
