# Supplementary material for: Trabectedin Enhances the Antitumor Effects of IL-12 in Triple-Negative Breast Cancer
Source: Cancer Immunol Res. 2025 Jan 7;13(4):560–76. doi: 10.1158/2326-6066.CIR-24-0775 (PMC11962391; doi:10.1158/2326-6066.CIR-24-0775)
Supplement: Supplementary Figure S5 [file cir-24-0775_supplementary_figure_s5_supps5.pdf]

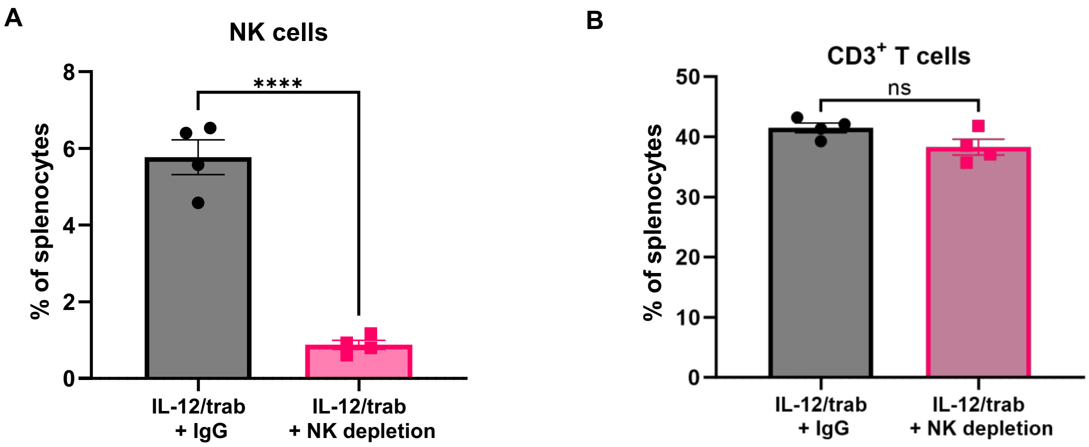

Supplementary Figure S5. Efficacy of anti-asialo-GM1 depletion antibody. (A) NK cell ($CD3^-$ $CD49b^+$) levels in the spleens of mice treated with IL-12+trabectedin with or without anti-asialo-GM1. (B) $CD3^+$ T cell levels in the spleens of mice treated with IL-12+trabectedin with or without anti-asialo-GM1. Statistical analyses were performed using two-tailed unpaired student's t-tests. Data represent mean ± SEM. ****$p<0.0001$.
