# Supplementary material for: Trabectedin Enhances the Antitumor Effects of IL-12 in Triple-Negative Breast Cancer
Source: Cancer Immunol Res. 2025 Jan 7;13(4):560–76. doi: 10.1158/2326-6066.CIR-24-0775 (PMC11962391; doi:10.1158/2326-6066.CIR-24-0775)
Supplement: Supplementary Figure S6 [file cir-24-0775_supplementary_figure_s6_supps6.pdf]

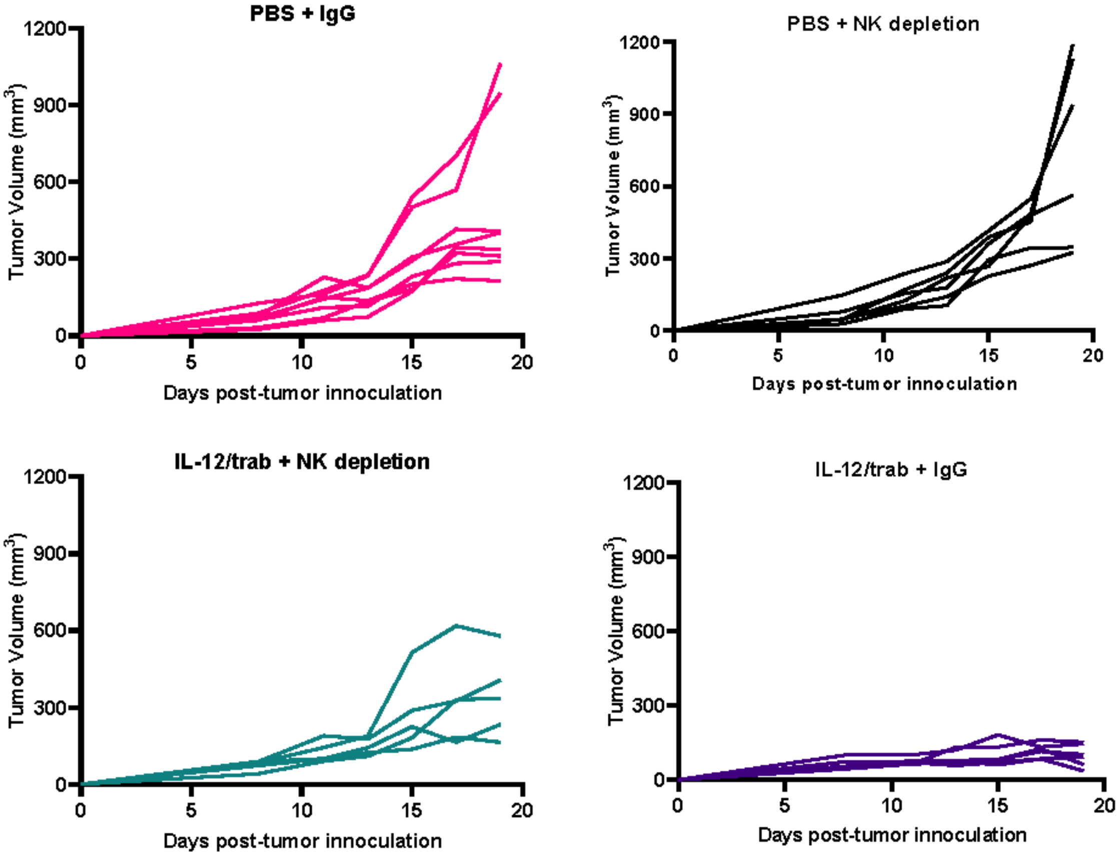

PBS + IgG

Supplementary Figure S6. Individual tumor growth curves highlight essentialness of NK cells to IL-12 and trabectedin therapy anti-tumor efficacy. (A) Spider plots of individual tumor growth curves throughout treatment from mice in **Fig. 5A** with and without NK cell depletion prior to IL-12 and trabectedin administration (n=5-8).
