# Supplementary material for: Trabectedin Enhances the Antitumor Effects of IL-12 in Triple-Negative Breast Cancer
Source: Cancer Immunol Res. 2025 Jan 7;13(4):560–76. doi: 10.1158/2326-6066.CIR-24-0775 (PMC11962391; doi:10.1158/2326-6066.CIR-24-0775)
Supplement: Supplementary Figure S8 [file cir-24-0775_supplementary_figure_s8_supps8.pdf]

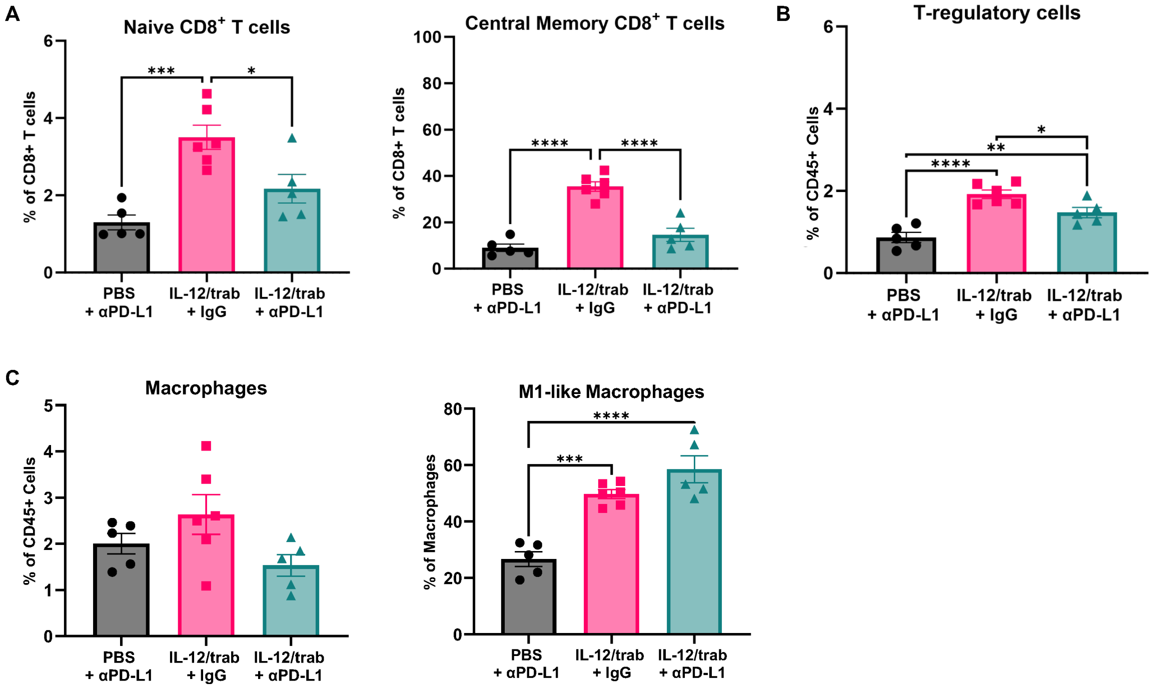

Supplementary Figure S8. Phenotypic changes in T cells and macrophages with anti-PD-L1 therapy. Percentage of (A) naïve ($CD62L^{+}CD44^{-}$), central memory ($CD62L^{+}CD44^{+}$) CD8+ T cells and (B) T-regulatory cells present in spleens from mice treated with anti-PD-L1 therapy, IL-12 and trabectedin therapy or the combination (n=5-6). (C) Percentage of total macrophages (left) and the proportion of M1-like macrophages (right) in spleens from mice treated with anti-PD-L1 therapy, IL-12 and trabectedin therapy or the combination (n=5-6). Statistical analyses were performed using ANOVA with Tukey's multiple comparisons test. Data represent mean ± SEM. *p<0.05, **p<0.01, ***p<0.001 ****p<0.0001.
