# Supplementary material for: Trabectedin Enhances the Antitumor Effects of IL-12 in Triple-Negative Breast Cancer
Source: Cancer Immunol Res. 2025 Jan 7;13(4):560–76. doi: 10.1158/2326-6066.CIR-24-0775 (PMC11962391; doi:10.1158/2326-6066.CIR-24-0775)
Supplement: Supplementary Table S1 [file cir-24-0775_supplementary_table_s1_suppst1.pdf]

Mass cytometry panel			
**Antibody**	**Mass**	**Clone**	**Vendor**
Ter119	89	TER-119	Biolegend
CD45	115	30-F11	Biolegend
GAPDH	139	6C5	Thermo Fisher
Ly6G	141	1A8	Biolegend
CD11c	142	N418	Biolegend
CD11b	143	M1/70	Biolegend
I-A/I-E	144	M5/114.15.2	Biolegend
CD19	149	6D5	Biolegend
Ly6C	150	HK1.4	Biolegend
CD25	151	PC61	Biolegend
CD317	152	927	Biolegend
B220	153	RA3-6B2	Biolegend
FceR1a	154	43890	Biolegend
CD69	155	H1.2F3	Biolegend
CD137	159	17B5	Biolegend
CD103	160	2 E 7	Biolegend
CD335	161	29A1	Biolegend
CD49b	162	DX5	Biolegend
CD107a	163	1D4B	Biolegend
CD44	164	IM7	Biolegend
pRB	165	J112-906	BD Biosciences
CD170/Siglec-F	166	E50-2440	Biolegend
CD206	167	C068C2	Biolegend
FoxP3	171	FJK-16s	eBioscience
F4/80	168	BM8	Biolegend
CD4	172	RM4-5	Biolegend
CD8a	173	53–6.7	Biolegend
CD3e	174	145-2C11	Biolegend
CD62L	176	MEL-14	Biolegend
IR-DNA	191	N/A	Fluidigm

Supplementary Table S1. Mass cytometry antibody panel.
