# Supplementary material for: Trabectedin Enhances the Antitumor Effects of IL-12 in Triple-Negative Breast Cancer
Source: Cancer Immunol Res. 2025 Jan 7;13(4):560–76. doi: 10.1158/2326-6066.CIR-24-0775 (PMC11962391; doi:10.1158/2326-6066.CIR-24-0775)
Supplement: Supplementary Table S2 [file cir-24-0775_supplementary_table_s2_suppst2.pdf]

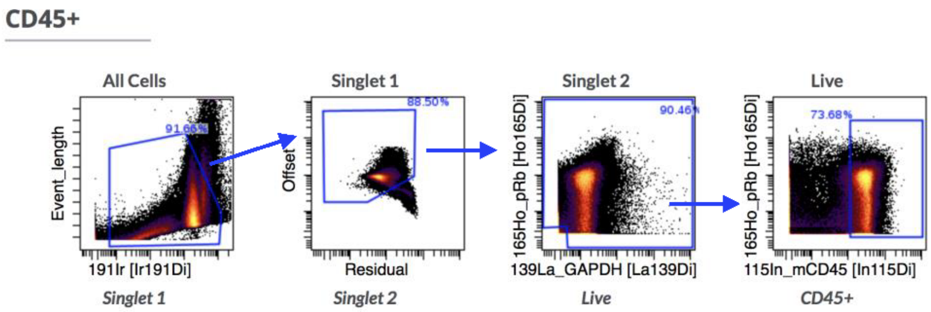

Cell population	Phenotype
**NK cells**	$CD45^+CD3^-CD49b^+NKp46^+$
**CD8+ T cells**	$CD45^+CD3^+CD8^+$
**CD4+ T cells**	$CD45^+CD3^+CD4^+$
**Naïve T cells**	$CD45^+CD3^+CD4/8^+CD44^-CD62L^+$
**Effector T cells**	$CD45^+CD3^+CD4/8^+CD44^+CD62L^-$
**Central memory T cells**	$CD45^+CD3^+CD4/8^+CD44^+CD62L^+$
**Tregs**	$CD45^+CD3^+CD4^+FoxP3^+CD25^+$
**B cells**	$CD45^+CD19^+B220^+$
**Macrophages**	$CD45^+CD11b^+F4/80^+$
**M1-like macrophages**	$CD45^+CD11b^+F4/80^+MHCII^+$
**M2-like macrophages**	$CD45^+CD11b^+F4/80^+CD206^+$
**Dendritic cells (DCs)**	
**cDC**	$CD45^+CD11b^-CD11c^+MHCII^+$
**cDC1**	$CD45^+CD11b^-CD11c^+MHCII^+CD103^+$
**mDC**	$CD45^+CD11b^+CD11c^+MHCII^+$
**pDC**	$CD45^+CD11b^-CD11c^+CD317^+$
**MDSC**	
**M-MDSC**	$CD45^+CD3^-CD11b^+Ly6C^{hi}Ly6G^-$
**PMN-MDSC**	$CD45^+CD3^-CD11b^+Ly6C^{low}Ly6G^+$

Supplementary Table S2. Mass cytometry live cell gating and immune cell population phenotypes.
