# Supplementary material for: Trabectedin Enhances the Antitumor Effects of IL-12 in Triple-Negative Breast Cancer
Source: Cancer Immunol Res. 2025 Jan 7;13(4):560–76. doi: 10.1158/2326-6066.CIR-24-0775 (PMC11962391; doi:10.1158/2326-6066.CIR-24-0775)
Supplement: Supplementary Table S3 [file cir-24-0775_supplementary_table_s3_suppst3.pdf]

Panel ID	Protein ID	Probe ID
Cell Death	BAD	DPROT_00246.1
Cell Death	BCLXL	DPROT_00248.1
Cell Death	BIM	DPROT_00254.1
Cell Death	Cleaved Caspase 3	DPROT_00473.1
Cell Death	PARP	DPROT_00253.1
Cell Death	Perforin	DPROT_00475.1
Cell Death	gamma-H2AX	DPROT_00338.1
Cell Death	p21	DPROT_00474.1
Cell Death	p53	DPROT_00477.1
Immune Activation Status	CD27	DPROT_00089.1
Immune Activation Status	CD86	DPROT_00273.1
Immune Activation Status	CD127/IL7RA	DPROT_00036.1
Immune Activation Status	CD40	DPROT_00087.1
Immune Activation Status	CD40L	DPROT_00088.1
Immune Activation Status	CD44	DPROT_00261.1
Immune Activation Status	ICOS	DPROT_00086.1
Immune Cell Profiling	GZMB	DPROT_00079.1
Immune Cell Profiling	Rb IgG	DPROT_00001.1
Immune Cell Profiling	Rt IgG2a	DPROT_00063.1
Immune Cell Profiling	Rt IgG2b	DPROT_00064.1
Immune Cell Profiling	GAPDH	DPROT_00020.1
Immune Cell Profiling	Histone H3	DPROT_00005.1
Immune Cell Profiling	S6	DPROT_00069.1
Immune Cell Profiling	CD11b	DPROT_00068.1
Immune Cell Profiling	CD11c	DPROT_00072.1
Immune Cell Profiling	CD19	DPROT_00073.1
Immune Cell Profiling	CD3e	DPROT_00074.1
Immune Cell Profiling	CD4	DPROT_00075.1
Immune Cell Profiling	CD45	DPROT_00076.1
Immune Cell Profiling	CD8a	DPROT_00078.1
Immune Cell Profiling	CTLA4	DPROT_00018.1
Immune Cell Profiling	F4/80	DPROT_00071.1
Immune Cell Profiling	Fibronectin	DPROT_00081.1
Immune Cell Profiling	Ki-67	DPROT_00070.1
Immune Cell Profiling	MHC II	DPROT_00067.1
Immune Cell Profiling	PD-1	DPROT_00065.1
Immune Cell Profiling	PD-L1	DPROT_00080.1
Immune Cell Profiling	PanCk	DPROT_00022.1
Immune Cell Profiling	SMA	DPROT_00023.1
Immune Cell Typing	BatF3	DPROT_00351.1
Immune Cell Typing	CD14	DPROT_00091.1
Immune Cell Typing	CD163	DPROT_00052.1
Immune Cell Typing	CD28	DPROT_00260.1
Immune Cell Typing	CD31	DPROT_00104.1
Immune Cell Typing	CD34	DPROT_00048.1
Immune Cell Typing	FOXP3	DPROT_00090.1
Immune Cell Typing	Ly6G/Ly6C	DPROT_00085.1
IO Drug Target	Tim-3	DPROT_00083.1
IO Drug Target	B7-H3	DPROT_00032.1
IO Drug Target	GITR	DPROT_00035.1
IO Drug Target	LAG3	DPROT_00082.1
IO Drug Target	OX40L	DPROT_00028.1
IO Drug Target	VISTA	DPROT_00084.1
MAPK Signaling	p38 MAPK	DPROT_00485.1
MAPK Signaling	BRAF	DPROT_00226.1
MAPK Signaling	EGFR	DPROT_00132.1
MAPK Signaling	MEK1	DPROT_00484.1
MAPK Signaling	Phospho-JNK (T183/Y185)	DPROT_00229.1
MAPK Signaling	Phospho-MEK1 (S217/S221)	DPROT_00230.1
MAPK Signaling	Phospho-p44/42 MAPK ERK1/2 (T202/Y204)	DPROT_00232.1
MAPK Signaling	Phospho-p90 RSK (T359/S363)	DPROT_00235.1
MAPK Signaling	p44/42 MAPK ERK1/2	DPROT_00234.1
MAPK Signaling	pan-RAS	DPROT_00222.1
PI3K/AKT Signaling	PLCG1	DPROT_00241.1
PI3K/AKT Signaling	MET	DPROT_00481.1
PI3K/AKT Signaling	Pan-AKT	DPROT_00318.1
PI3K/AKT Signaling	Phospho-AKT1 (S473)	DPROT_00236.1
PI3K/AKT Signaling	Phospho-AMPK-alpha (T172)	DPROT_00482.1
PI3K/AKT Signaling	Phospho-GSK3A (S21)/Phospho-GSK3B (S9)	DPROT_00334.1
PI3K/AKT Signaling	Phospho-PRAS40 (T246)	DPROT_00242.1
PI3K/AKT Signaling	Phospho-S6 (S235/S236)	DPROT_00315.1

Supplementary Table S3. NanoString digital spatial profiling protein panel.
