# Supplementary material for: Trabectedin Enhances the Antitumor Effects of IL-12 in Triple-Negative Breast Cancer
Source: Cancer Immunol Res. 2025 Jan 7;13(4):560–76. doi: 10.1158/2326-6066.CIR-24-0775 (PMC11962391; doi:10.1158/2326-6066.CIR-24-0775)
Supplement: Supplementary Table S4 [file cir-24-0775_supplementary_table_s4_suppst4.pdf]

A **Control vs IL-12+trabectedin CD45⁺**

Protein	Change	Fold change (log2)	Adjusted p-value
**Ki-67**	Decreased	-0.58	0.0008
**CD31**	Decreased	-0.82	0.0008
**CD11c**	Decreased	-0.50	0.0031
**SMA**	Decreased	-1.03	0.0121
**CD40**	Decreased	-0.77	0.0293
**MET**	Decreased	-0.47	0.0425

B **IL-12 vs IL-12+trabectedin CD45⁺**

Protein	Change	Fold change (log2)	Adjusted p-value
**Ki-67**	Decreased	-0.74	5.59E-06
**CD27**	Decreased	-1.23	7.45E-05
**SMA**	Decreased	-1.40	0.000174473
**Phospho-p90 RSK**	Decreased	-1.01	0.000478734
**CTLA4**	Decreased	-0.73	0.001609566
**CD11c**	Decreased	-0.49	0.001613442
**Phospho-S6**	Decreased	-1.15	0.001815629
**GZMB**	Increased	1.28	0.005979624
**Fibronectin**	Increased	1.52	0.007467886
**Ly6G/Ly6C**	Increased	1.87	0.007624948
**PLCG1**	Decreased	-0.56	0.009726871
**CD11b**	Increased	1.31	0.012107207
**Phospho-AKT1**	Decreased	-0.70	0.013332676
**Pan-AKT**	Decreased	-0.55	0.018590003
**FOXP3**	Decreased	-0.76	0.019213635
**OX40L**	Decreased	-0.62	0.021202436
**Phospho-MEK1**	Decreased	-0.78	0.030908959
**Perforin**	Increased	1.16	0.036208339
**Phospho-AMPK-alpha**	Decreased	-0.70	0.036208339
**p38 MAPK**	Decreased	-0.37	0.036515468
**CD40L**	Increased	0.91	0.055558685

C **Trabectedin vs IL-12+trabectedin CD45⁺**

Protein	Change	Fold change (log2)	Adjusted p-value
**CD8a**	Increased	0.80	5.31E-05
**ICOS**	Increased	1.28	5.31E-05
**Ki-67**	Decreased	-0.52	0.001961987
**LAG3**	Increased	1.08	0.03597907
**CD163**	Decreased	-1.50	0.03597907
**BatF3**	Increased	0.41	0.03597907
**CD3e**	Increased	0.43	0.043539208
**Ly6G/Ly6C**	Increased	1.59	0.043539208
**PD-1**	Increased	0.51	0.044061265

Supplementary Table S4. Significantly differentially expressed proteins in CD45⁺ tumor ROIs across treatment groups.
